# Combining and Comparing Coalescent, Distance and Character-Based Approaches for Barcoding Microalgaes: A Test with *Chlorella*-Like Species (Chlorophyta)

**DOI:** 10.1371/journal.pone.0153833

**Published:** 2016-04-19

**Authors:** Shanmei Zou, Cong Fei, Jiameng Song, Yachao Bao, Meilin He, Changhai Wang

**Affiliations:** Jiangsu Provincial Key Laboratory of Marine Biology, College of Resources and Environmental Science, Nanjing Agricultural University, Nanjing 210095, PR China; Queensland University of Technology, AUSTRALIA

## Abstract

Several different barcoding methods of distinguishing species have been advanced, but which method is the best is still controversial. *Chlorella* is becoming particularly promising in the development of second-generation biofuels. However, the taxonomy of *Chlorella–*like organisms is easily confused. Here we report a comprehensive barcoding analysis of *Chlorella-*like species from *Chlorella*, *Chloroidium*, *Dictyosphaerium* and *Actinastrum* based on *rbcL*, ITS, *tufA* and 16S sequences to test the efficiency of traditional barcoding, GMYC, ABGD, PTP, P ID and character-based barcoding methods. First of all, the barcoding results gave new insights into the taxonomic assessment of *Chlorella-*like organisms studied, including the clear species discrimination and resolution of potentially cryptic species complexes in *C*. *sorokiniana*, *D*. *ehrenbergianum* and *C*. *Vulgaris*. The *tufA* proved to be the most efficient barcoding locus, which thus could be as potential “specific barcode” for *Chlorella-*like species. The 16S failed in discriminating most closely related species. The resolution of GMYC, PTP, P ID, ABGD and character-based barcoding methods were variable among *rbcL*, ITS and *tufA* genes. The best resolution for species differentiation appeared in *tufA* analysis where GMYC, PTP, ABGD and character-based approaches produced consistent groups while the PTP method over-split the taxa. The character analysis of *rbcL*, ITS and *tufA* sequences could clearly distinguish all taxonomic groups respectively, including the potentially cryptic lineages, with many character attributes. Thus, the character-based barcoding provides an attractive complement to coalescent and distance-based barcoding. Our study represents the test that proves the efficiency of multiple DNA barcoding in species discrimination of microalgaes.

## Introduction

*Chlorella* (Trebouxiophyceae, Chlorophyta), single-celled green algae, is one of the most famous microalgae genus worldwide that grow in marine, freshwater or edaphic habitats. *Chlorella* could be used as powerful ‘superfoods’, and is significant in the development of second-generation biofuels and medical treatments [1–4]. Nevertheless, the taxonomic assignment of *Chlorella* is easily confused since there are no obvious structural features among species or some of observable characteristics are variable within species. After the type species are identified, more than 100 *Chlorella* species have been described [5–9]. For a long time, numerous studies focusing on morphological characters, ultrastructural composition of the cell wall, biochemical and physiological characters and molecular phylogenetic characteristics have been carried out to revise the system of *Chlorella* [6,9–25]. New *Chlorella*-related species and genera are often discovered in recent studies [4,26–30]. Most notably, *Chlorella* species are recognized as members of Chlorophyceae and Trebouxiophyceae. Based on the biochemical and molecular data, *Chlorella* is shown that it consists of only five “true” *Chlorella* species [9,18,20]. Darienko et al. [22] propose to transfer all *Chlorella*-like strains that have been identified as *Chlorella saccharophila* and *Chlorella ellipsoidea* to the genus *Chloroidium* in the so-called *Watanabea* clade. According to recent molecular studies, species that have typical *Chlorella* morphology are assigned to the family Chlorellaceae (Trebouxiophycean) that is divided into *Chlorella*-clade and the *Parachlorella*–clade [20,31,32]. Based on SSU- and ITS rDNA sequences and light microscopic observations, Bock et al. [25] detect six lineages of *Dictyosphaerium*-like strains that are closely related to *Chlorella vulgaris* and describe several new species. Consequently, high levels of cryptic diversity found within *Chlorella* and the polyphyletic characters between *Chlorella* and *Dictyosphaerium* results in fundamental taxonomic revision of these organisms, e.g. the description of many new species and genera [20,23–25,32,33]. All these studies indicate that the classification of *Chlorella* is still very confused and it is urgent to revise the genus. However, the identification of *Chlorella–*like taxa in the species level is still unclear. It is still unknown how many species are actually included in *Chlorella*. Moreover, most previous molecular studies generally focus on the phylogenic analysis of *Chlorella-*related clades based on 18S, ITS or SSU gene data [21,31–33]. The molecular taxonomic identification of *Chlorella*-like microalgaes often analyzes limited gene loci or samples. For example, the taxonomic reassessment of *Chlorella* by molecular signatures (barcodes) [24] is just based on the ITS. Therefore, numerous cryptic species within *Chlorella-*like organisms may be overlooked. To recover the hidden diversity, more molecular markers that have sufficient nucleotide diversity, low saturation and a simple alignment process should be used for taxonomic identification [32].

DNA barcoding is the most promising approach for species identification and detection of cryptic species and potentially new species, particularly for the microbial communities [34–44]. The traditional DNA barcoding [34], including the monophyly and distance-based methods, are originally used for DNA barcoding. The distance method relies on the ‘barcoding gap’ and the monophyly method can reconstruct the evolutionary histories of character traits [34,35,45]. However, for some taxonomic groups, it is impossible to identify the specimens based on intraspecific variation vs. interspecific divergence. For example, in plants, the species discrimination is not always accurate using the ‘barcoding gap’, e.g. [46–49], and in some cases, species identification is possible even if a ‘barcoding gap’ is absent [50]. The monophyly-based DNA barcoding approach has the drawback that relates to the use of hierarchical methods for identification [51–60]. On the other hand, the phylogenetic tree is often used for flagging species.

Recently, several different methods have been put forward for distinguishing species [61]. The generalized mixed Yule–coalescent model sets a threshold to delineate evolutionary significant units (ESUs) akin to species [62–64]. The P ID (Liberal) method of species delimitation is advanced for the exploration of species boundaries [65], which allows differing species boundary hypotheses to be investigated by enabling the user to a priori assign taxa to putative species groups on a phylogenetic tree. The poisson tree process (PTP) model is another tree-based method that distinguishes specimens in both populations and species level using coalescence theory [66]. Automatic Barcode Gap Discovery (ABGD), a new distance method, can assign the sequences into potential species based on the barcode gap whenever the divergence within the same species is smaller than that among organisms from different species [67].

The character-based barcoding approach has recently been proved useful in species identification and cryptic species revelation of some organisms (including some plants) [39,40–41,50,68–72]. It is based on the concept that members of a given taxonomic group have the same diagnostic characters that are absent from comparable groups [69,73]. The character-based approach has the logical advantage that it will fail to diagnose the specimens when diagnostic character data are lacking, in comparison with using distances. With the development of DNA barcoding, it seems that combination of improved species-level phylogenetic trees and new statistical methods that evaluate quantitative character states will greatly help us to understand the biodiversity patterns [74,75]. In this context, combination of multiple DNA barcoding approaches may be more effective to reveal cryptic biodiversity. However, the character-based barcoding approach is not yet commonplace in barcoding practice. So far, few studies about barcoding microalgaes have been performed using character-based methods. On the contrary, most molecular taxonomic identification of algae is just based on the phylogenetic trees or genetic distance, including DNA barcoding of marine green macroalgae [76], freshwater green algae [77] and some *Chlorella-*related samples [24].

Besides the barcoding approaches, the efficient ‘DNA barcodes’ also play an important role in successful species identification across a wide range of taxonomic groups. Due to a much slower mutation rate the cytochrome *c* oxidase 1 (CO1) sequence which has been proved efficient in barcoding animals does not discriminate most plants [78,79]. Although in red and brown algae and some diatoms, the 5’ end of COI (COI-5P) provides resolution at the species level [79–84], it is often unsuccessfully amplified in green algae, despite extensive primer testing [76,77]. The presence of introns within COI [85–88] may be the largest obstacle to developing the COI-5P as a suitable DNA barcode marker for green algae. For land plants the core DNA barcodes are portions of two plastid coding genes (*rbcL* and *matk*) [89]. However, the candidate loci fail in eliminating the disadvantages of current DNA barcoding for plants [90]. Efficient ‘DNA barcodes’ for plants are still unknown. Moreover, since *matK* is absent in algae it is urgently needed to select the specific barcodes for taxonomic group of algae, especially for microalgaes. Recently, a new concept, the ‘specific barcode’ is proposed, which refers to a fragment of DNA sequence that can enable species identification within a given taxonomic group (e.g. a genus or family) by sufficiently high mutation rate [91]. ‘Specific barcodes’ for plants can assist species-level identifications. Presently the *rbcL* gene (encodes the large subunit of Rubisco), *tufA* gene (encoding elongation factor) and ITS (internal transcribed spacer region) have been proved useful in discriminating some microalgae species, e.g. [76,77,92]. Thus, they could be candidates as ‘specific barcodes’ for green algae.

In this study we present a comprehensive DNA barcode analysis (traditional barcoding, GMYC, P ID, PTP, ABGD and character-based approaches) of *Chlorella*-like species from *Chlorella*, *Chloroidium*, *Actinastrum* and *Dictyosphaerium*, based on four gene loci *rbcL*, *tufA*, ITS (ITS1-5.8S-ITS2) and 16S. Publicly available sequences were added to the newly obtained sequences from this study to better evaluate identification success among the organisms. Sequences from all the genes are used: (i) to identify *Chlorella*-like taxa and reveal the possible existence of cryptic species (ii) to evaluate the efficiency of coalescent, distance and character-based barcoding approaches in retrieving the taxon identities of this morphologically complex microalgaes.

## Materials and Methods

### Ethics Statement

No specific permits were required for the described field studies. The field studies did not involve endangered or protected species. No specific permissions were required for the locations. The locations are not privately-owned or protected in any way.

### Algal sampling, culturing and morphological identification

The *Chlorella*-like green microalgaes studied were from the genera *Chlorella*, *Chloroidium*, *Actinastrum* and *Dictyosphaerium*, most of which were *Chlorella* strains. A total of 176 *Chlorella*-like samples were analyzed. The collection spots covered marine, freshwater, north pole and terrestrial areas. The procedure for clone isolation followed Andersen [93]. The nonaxenic strains were grown in 250 mL flask containing 200 mL liquid at an irradiance of 40 umol m^-2^ s^-1^ with 14:10 h light: dark cycle at 20°C. Some strains were obtained from different Culture Collections, e.g. Austin, Texas, Waller Creek at University Campus, USA. A detailed list of taxa studied, was provided in S1 Table.

The samples collected in this study were first identified based on the available morphological characters. Specimens that could not to be assigned to binomial names were just labeled as unknowns.

### DNA Extraction, Amplification and Sequencing

DNA was extracted using the Qiagen DNEasy Plant Extraction kit (Qiagen Inc., Valencia, CA, USA) following the instructions given by the manufacturer. The *rbcL*, *tufA*, ITS and 16S barcode regions were amplified and sequenced from most species using universal primers or primers designed in this study (S2 Table) [24, 94–96]. PCR reactions for all barcode regions were carried out in a total volume of 25 μL, using 2×Taqman PCR MasterMix. PCR conditions for all primer sets were as follows: 95°C for 3 min, primer-specific annealing temperatures for 45s, 72°C for 1 min; 35 cycles of 95°C for 30 s, primer-specific annealing temperatures for 45s, 72°C for 1 m, with a final extension of 72°C for 1 min. Then the PCR products were sequenced on an ABI 3730XL (Applied Biosystems).

### Sequence alignment

Forward and reverse sequences of each region were edited in Sequencher (Gene Codes Corporation), and a set of publicly available sequences from Genbank was added. All *rbcL*, ITS, 16S and *tufA* sequences were aligned using MAFFT 6.717 [97] and trimmed to a region 1158 nucleotides, 1016–1300 nucleotides, 315–429 nucleotides and 783 nucleotides in length respectively.

### Traditional barcoding analysis

#### Phylogenetic reconstruction

Neighbour joining trees of *rbcL*, *tufA*, ITS and 16S sequences were constructed based on Kimura 2-parameter (K2P) distance model as recommended by Hebert et al. [34–35] in MEGA 5.0 [98] with bootstrap values (1000 replications). Since the identification of *Chlorella*-like species was difficult by morphological characters, the NJ trees were first used to flag species for character-based barcoding analysis.

For Bayesian analysis of each gene, the jModeltest v.0.1.1 [99] was used to estimate the best substitution model using Akaike Information Criterion (AIC). The most appropriate models for *rbcL*, ITS, 16S and *tufA* were GTR+G, GTR+ G, TVMef+I+G and GTR +G respectively. As described in detail previously [41], the Bayesian analysis were conducted in MRBAYES 3.1.2 [100]. The maximum-likelihood (ML) search was performed using PHYML 3.0 [101].

#### Distance analyses

Genetic distances were ascertained using MEGA 5.0 [98] and the Distance Summary applications of the BOLD website, with the K2P model [37]. To assess the barcoding gap effectiveness, the analyses of intra- and interspecific divergences were conducted among the taxa assignments based on multiple method-based barcoding analyses.

### GMYC species delimitation

Using BEAST [102,103], a linearised Bayesian phylogenetic tree was first calculated employing a Yule pure birth model [104] (Gernhard 2008) tree prior. Settings in BEAUTi v. 1.7.1 were: substitution models for each gene, empirical base frequencies, four gamma categories, all codon positions partitioned with unlinked base frequencies and substitution rates. An uncorrelated relaxed lognormal clock model was used with rate estimated from the data and ucldmean parameter with uniform prior to value 0 as a lower and 10 as an upper boundary. All other settings were left as defaults. The length of MCMC chain was 40 000 000 sampling every 4000. All BEAST runs were executed in Bioportal [105], and the ESS values and trace files of runs were evaluated in Tracer v1.5.0. Two independent runs were merged using Log-Combiner v1.7.1 with 20% burn-in. Maximum clade credibility trees with a 0.5 posterior probability limit, and node heights of target tree were constructed in TreeAnnotator v1.7.1. Single-threshold GMYC analyses was conducted in R [106] using the APE [107] and SPLITS [108] packages.

### Poisson tree process model (PTP)

Since the ultrametric trees are not required as input this coalescent-based method is very fast. This method is implemented in a web server (http://species.h-its.org/).

### P ID (Liberal) species boundary delimitation

The Species Delimitation plugin [65] within Geneious Pro v5.5.4 (Biomatters; http://www.geneious.com) was investigated to assess species boundary hypotheses across the Bayesian gene tree. Geneious is a bioinformatics desktop software package produced by Biomatters Ltd (http://www.biomatters.com). P ID(Liberal) in Geneious, represents the probability of making a correct identification of an unknown specimen by measuring the genetic variation found within its putative species group and comparing that to the species group with which it is most likely to be confused [109]. Maximum Likelihood trees were inferred from *rbcL*, *tufA* and ITS datasets by employing PhyML 3.0 [101].

### Automatic Barcode Gap Discovery

The ABGD method is available at http://wwwabi.snv.jussieu.fr/public/abgd/. The *rbcL*, 16S, ITS and *tufA* complete sequence data were processed in ABGD using the K2P nucleotide substitution model. Prior for the maximum value of intraspecific divergence was set between 0.001 and 0.1, and 10 recursive steps within the primary partitions was defined by the first estimated gap. The gap width was set 1.0.

### Character-based DNA barcode analyses

Pure unique identifying characters, termed diagnostic characters or “characteristic attributes” (CAs) that distinguish a species from others, were determined using characteristic attribute organization system (CAOS) which comprises P-Gnome and P-Elf programs [66,110]. The CAOS algorithm could extract CAs for each clade at branching node within a guide tree [69]. In this study, the guide trees inferred from *rbcL*, ITS and *tufA* sequences were first produced using the programs PAUP v4.0b10 [111], and were incorporated into a NEXUS file containing *rbcL*, ITS and *tufA* sequence data respectively in MacClade [112]. Then the incorporated NEXUS datasets were conducted in CAOS system where the P-Gnome script was used to identify characters. Finally, the most variable character states were listed.

## Results

A total of 176 *Chlorella*-like samples from this study and publicly available data were analyzed. PCR amplification and sequencing were successful with the ITS locus in most of the samples (S1 Table). With the *rbcL*, 16S and *tufA* loci, there were more difficulties in amplification and in sequencing, especially for *tufA*. A total of 96 *rbcL*, 76 ITS, 86 16S and 66 *tufA* sequences of *Chlorella-*like samples and outgroups were analyzed (S1 Table). The accession numbers of newly obtained sequences submitted to the GenBank Barcode database were: KM514738-KM514804 for 16S, KM514805-KM514860 for ITS, KM514861-KM514917 for *rbcL* and KR154236-KR154291 for *tufA*.

### Traditional DNA barcoding

#### Phylogenetic analyses

**Fig 1:**
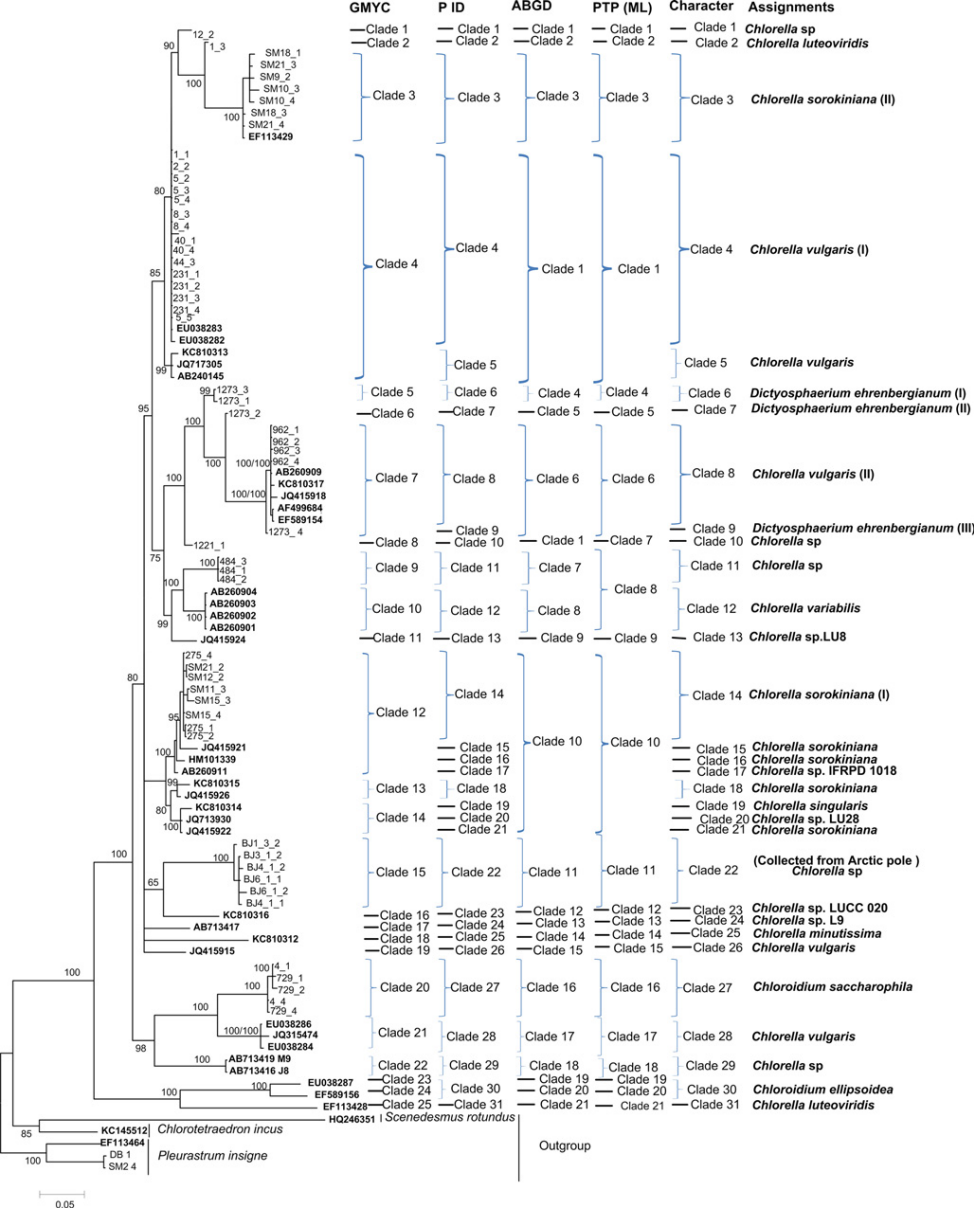
Bayesian phylogenetic tree for the *rbcL* gene. *Vertical bars on the right* indicate the clades detected by the coalescent-based GMYC, PID, PTP, the distance-based ABGD approach, the character-based CAOS and the final assignment. Posterior probabilities and NJ bootstrap values were included.

**Fig 2:**
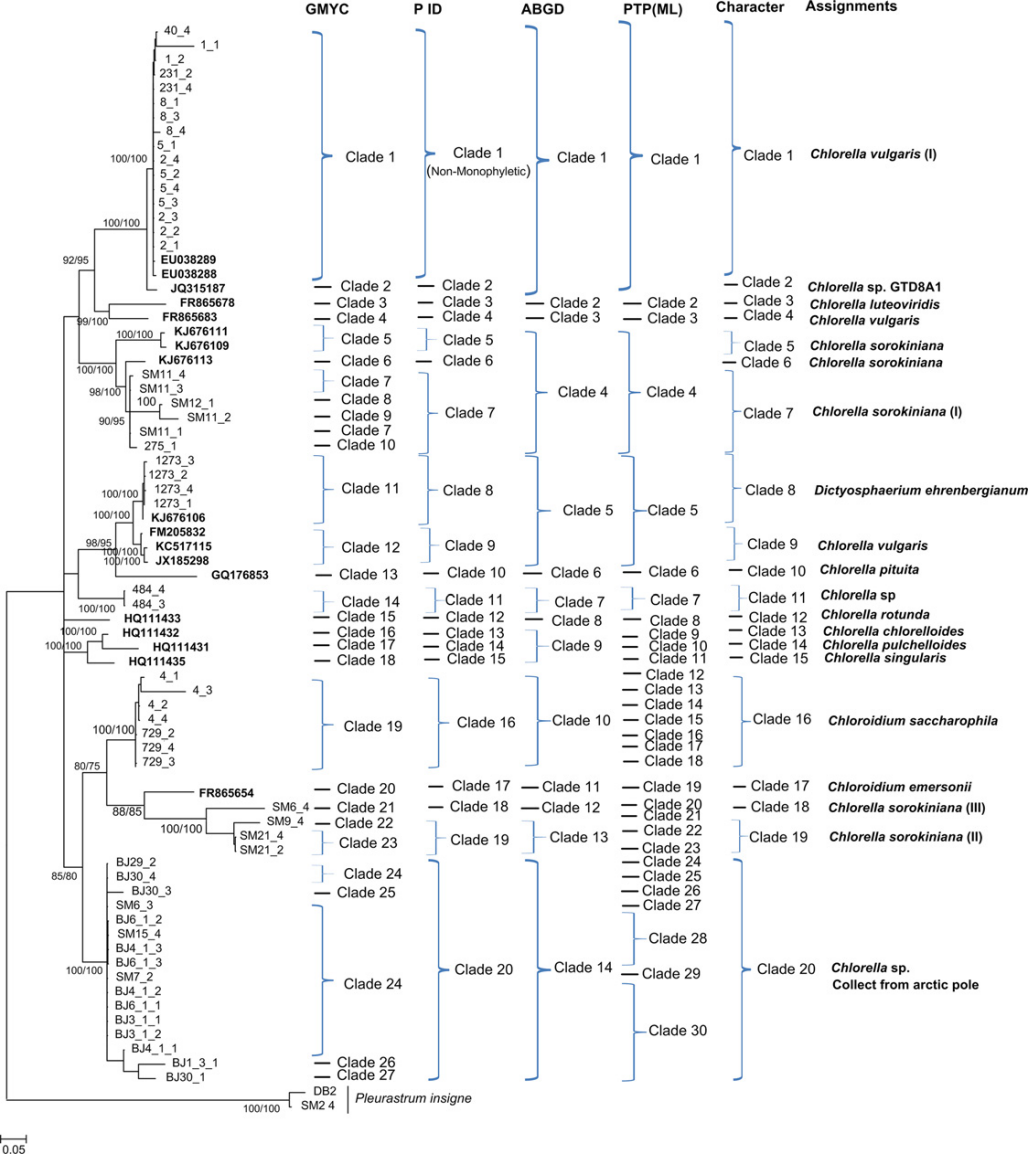
Bayesian phylogenetic tree for the ITS gene. Vertical bars on the right indicate the clades detected by the coalescent-based GMYC, PID, PTP, the distance-based ABGD approach, the character-based CAOS and the final assignment. Posterior probabilities and NJ bootstrap values were included.

**Fig 3:**
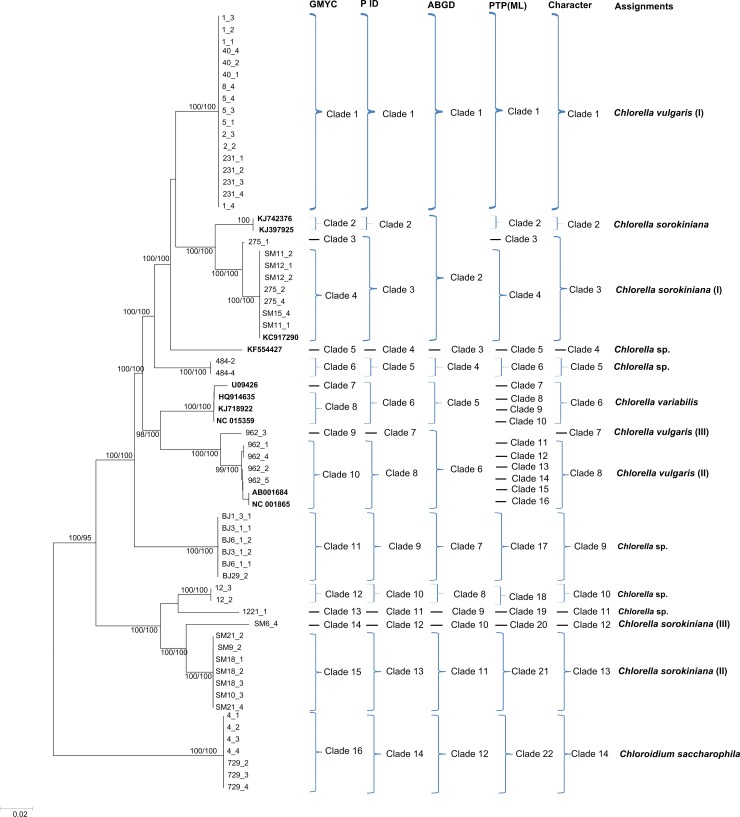
Bayesian phylogenetic tree for the *tufA* gene. *Vertical bars on the right* indicate the clades detected by the coalescent-based GMYC, PID, PTP, the distance-based ABGD approach, the character-based CAOS and the final assignment. Posterior probabilities and NJ bootstrap values were included.

Generally, the NJ, Bayesian and Maximum Likelihood trees of *rbcL*, ITS and *tufA* recovered consistent groups respectively (Figs 1–3 and S1 Fig, S2 Fig and S3 Fig). A total of 31, 20 and 14 monophyletic *Chlorella-*like clades were recovered in *rbcL*, ITS and *tufA* bayesian trees respectively (Figs 1–3), including the potentially cryptic lineages in *Chlorella sorokiniana*, *Dictyosphaerium ehrenbergianum* and *Chlorella vulgaris*, These recovered lineages would be further analyzed by GMYC, PTP, P ID, ABGD and character-based barcoding. Sequences of *C*. *sorokiniana*, *D*. *ehrenbergianum* and *C*. *vulgaris* fell into several distinct clades respectively in *rbcL*, ITS and *tufA* trees, which might be indicating potentially cryptic lineages (Figs 1–3). Thereinto, *C*. *sorokiniana* was divided into: five clades (I), (II), (KC810315, JQ415926), (JQ415921) and (HM101339) in *rbcL* tree (Fig 1); five clades (I), (II), (III), (KJ676111, KJ676109) and (KJ676113) in ITS tree (Fig 2); and four clades (I), (II), (III) and (KJ742376, KJ397925) in *tufA* tree (Fig 3). *C*. *vulgaris* was also divided into: five clades (I), (II), (EU038286, JQ315474, EU038284), (KC810313, JQ717305, AB240145) and (JQ415915) in *rbcL* tree (Fig 1); three clades (I), (FR865683) and (FM205832, KC517115, JX185298) in ITS tree (Fig 2); and three clades (I), (II) and (III) in *tufA* tree (Fig 3). *D*. *ehrenbergianum* was divided into three clades (I), (II) and (III) in *rbcL* tree (Fig 1). It was worth noting that all the samples collected from Arctic pole grouped together as a separate clade, but they still could not be identified to the specific taxa in the species level. In addition, most unknown samples were recovered as separate clades that did not group together with other species (Figs 1–3). The 16S NJ, Bayesian and Maximum trees, however, could not separate the closely related *Chlorella-*like samples, and the supports for the monophyletic clades were very low (S4 and S5 Figs). Therefore, the coalescent, distance and character assignments of 16S sequences were not analyzed in this study. It was apparent that the *tufA* phylogenetic trees recovered more well-supported monophyletic taxa in comparison with the *rbcL*, ITS and 16S phylogenies.

#### Distance analyses

**Fig 4:**
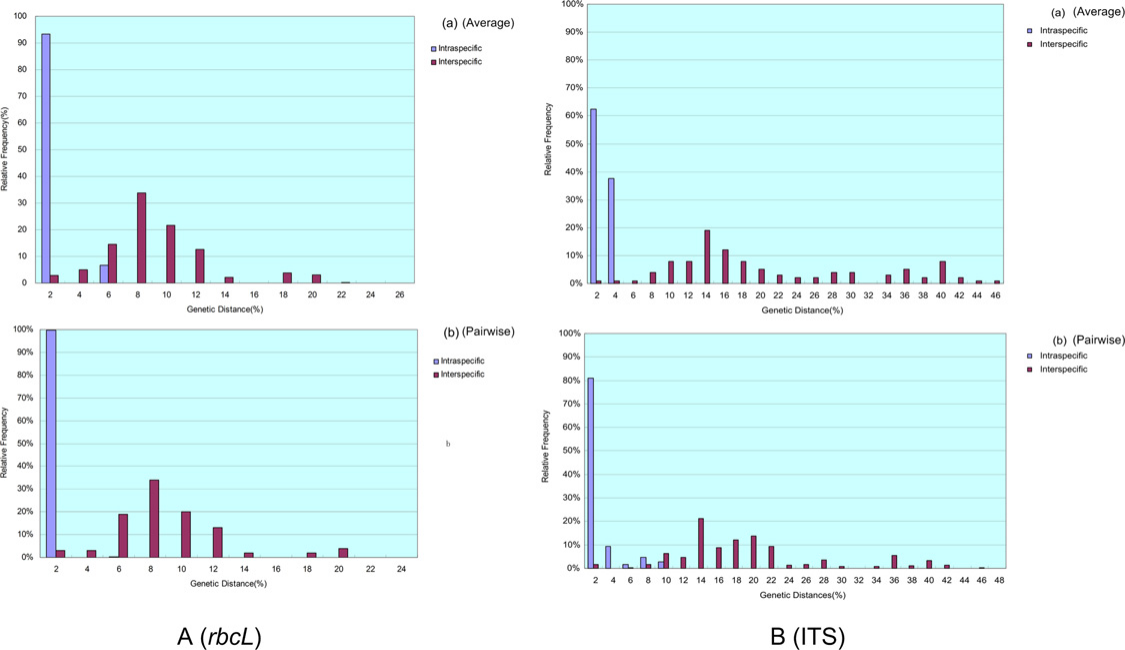
**A** (a): Histograms of intra- (in blue) and inter-specific (in red) average distances between *rbcL* sequences; (b): Histograms of intra- (in blue) and inter-specific (in red) pairwise distances between *rbcL* sequences. **B** (a): Histograms of intra- (in blue) and inter-specific (in red) average distances between ITS sequences; (b) Histograms of intra- (in blue) and inter-specific (in red) pairwise distances between ITS sequences.

**Fig 5:**
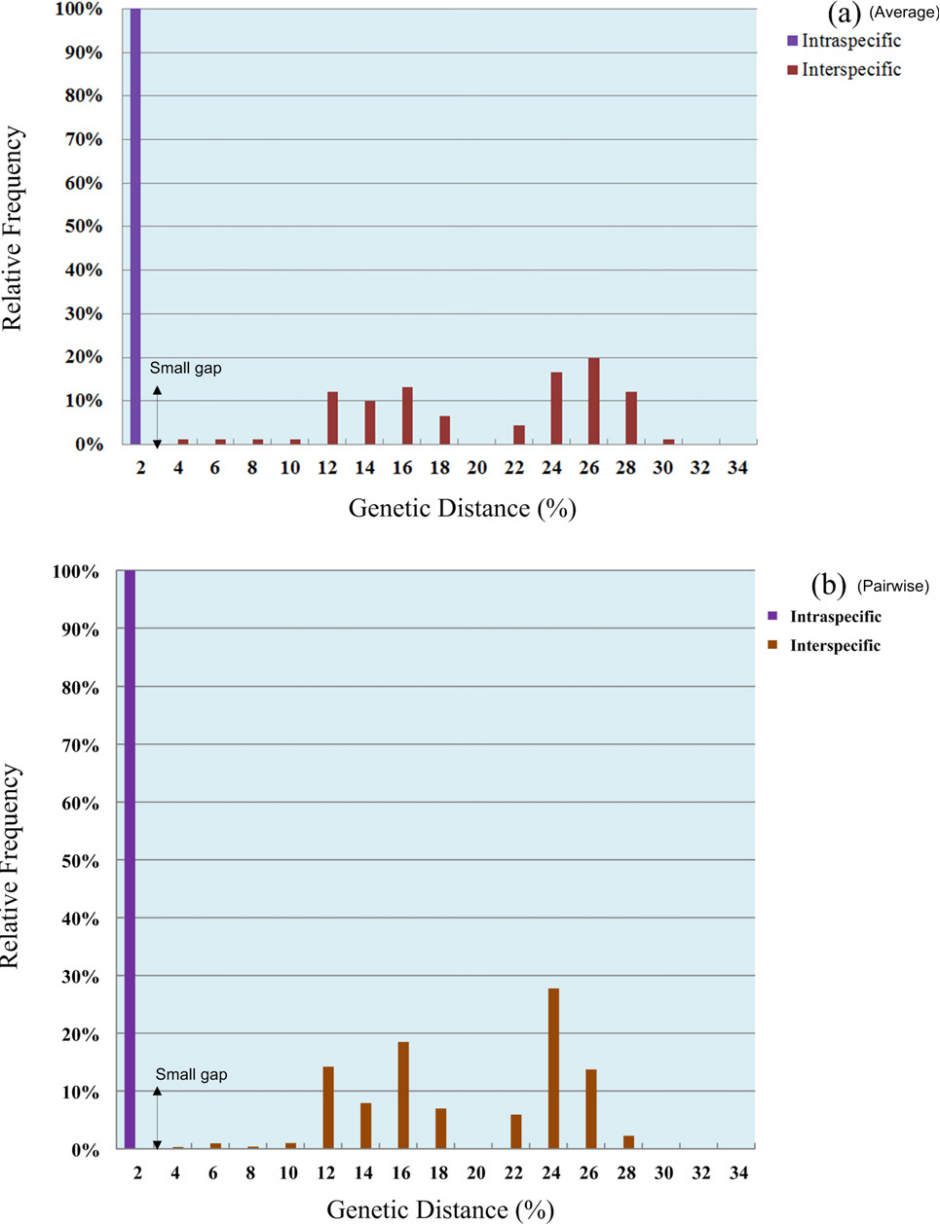
(a) Histograms of intra- (in blue) and inter-specific (in red) average distances between *tufA* sequences; (b) Histograms of intra- (in blue) and inter-specific (in red) pairwise distances between *tufA* sequences.

Based on the phylogenetic, GMYC, PTP, P ID, ABGD and character-based barcoding assignments of *rbcL*, ITS and *tufA* sequences, intra- and interspecific variation of above defined *Chlorella-*like assignments was conducted respectively (Figs 1–3), and the existence of DNA barcoding gap was tested. The results showed that the pairwise intraspecific distance of *rbcL* were from 0% to 4.2% with a mean of 0.51% while the pairwise interspecific distances was from 0% to 21.70%. The mean *rbcL* divergence among the possible cryptic lineages within *C*. *sorokiniana*, *D*. *ehrenbergianum* and *C*. *vulgaris* were from 1.3% to 9.8%, 2.3% to 5.4% and 0.9% to 9.6% respectively, which was higher than 0.51% (the mean intraspecific distance) (S3 Table). However, no clear barcoding-gap was found between the intra- and interspecific distances of *rbcL* sequences (Fig 4A). The ITS pairwise intraspecific distance ranged from 0% to 9.3% with a mean of 1.6% while the pairwise interspecific distances was from 0% to 45.1%. The mean ITS divergence among the separate cryptic lineages within *C*. *sorokiniana* and *C*. *vulgaris* ranged from 1.7%-36.6% and 13.4%-18.4% respectively, which were also higher than 1.6% (the mean intraspecific distance) (S4 Table). However, as with *rbcL*, there was apparent overlap between the intra- and interspecific distances of ITS sequences (Fig 4B). The *tufA* divergences among 14 *Chlorella-*like taxa in phylogenetic and character analysis were analyzed. The *tufA* pairwise intraspecific distance ranged from 0% to 1% with a mean of 0.1% while the pairwise interspecific distances was from 0% to 28.6%. The mean *tufA* divergence among the cryptic lineages *C*. *sorokiniana* (I),(II),(III),(KJ742376,KJ397925) and *C*. *vulgaris* (I),(II),(III), ranged from 7.9%-28.6% and 10.7%-16.1% respectively, which was also greatly higher than the mean intraspecific distance (0.1%) (S5 Table). Since all the intraspecific distance was lower than 2% (proposed as 10× rule by Hebert et al. [35]) and all the interspecific distance was higher than 2%, there was a small barcoding gap between the intra- and interspecific variation of *tufA* sequences (Fig 5).

### ABGD analysis

Based on the distance-based approach as implemented in the software ABGD, different groups as candidate species were produced for *rbcL*, ITS and *tufA* gene sequences. Generally, the ABGD analysis of *rbcL*, ITS and *tufA* produced fewer genetic groups than other barcoding methods (Figs 1–3). For *rbcL*, the ABGD analysis revealed 21 genetic groups when using restrictive values with priori genetic distance thresholds 0.77% (Fig 1 and S6 Fig). Most of the 21 groups were consistent with the ABGD analysis of ITS and *tufA* in which 14 groups and 12 groups were produced at a priori genetic distance thresholds of 1% and 3.59% respectively (Figs 2 and 3, S7 and S8 Figs). In all analyses, *C*. *vulgaris* and *C*. *sorokiniana* were split into several groups.

### GMYC species delimitation

The optimal threshold points obtained by the GMYC model for *rbcL*, ITS and *tufA* genes were shown in Figs 1–3, S9–S11 Figs, respectively. As a whole, the specimens studied were oversplitted by the GMYC model for *rbcL*, ITS and *tufA* genes in comparison with ABGD analysis (Figs 1–3). The results of the single threshold analysis for the *rbcL*, ITS and *tufA* gene suggested 25, 27 and 16 groups respectively, some of which consisted of single specimens. Especially in ITS analysis, the *C*. *sorokiniana* (I) and *C*. sp. Collect from Arctic pole were split into several groups.

### PTP-based identification

The resolution produced by bPTP approach was variable among *rbcL*, ITS and *tufA* genes (Figs 1–3). The maximum-likelihood identification produced better resolution than bayesian identification. For *rbcL*, it recognized 21 independent entities which were consistent with the groups revealed by ABGD analyses (Fig 1). However, for ITS and *tufA*, the taxa were all over split by PTP analysis than other methods (Figs 2 and 3).

### P ID-based identification

Based on the bayesian analysis, the tree-based hypotheses were reevaluated for species hypothesis testing. Most candidate species were recovered as monophyletic clades in P ID species boundary delimitation of *rbcL*, ITS and *tufA* genes except *Chlorella vulgaris* (I) which was not monophylic in *rbcL* analysis (Figs 1–3). The resolution produced by P ID method was generally consistent with the Character analysis. All delimited species of *rbcL*, ITS and *tufA* possessed a P ID (Liberal) value P>0.7 (S6–S8 Tables).

### Character-based identification

**Fig 6:**
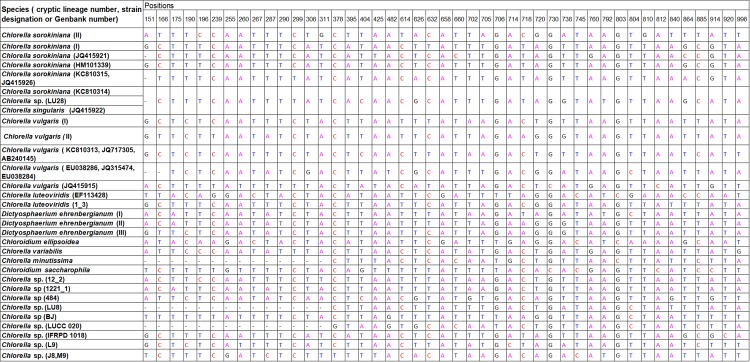
Combinations of diagnostic nucleotides for each of the 31 *Chlorella*-like taxa recovered in Fig 1. Nucleotide numbers refer to 45 selected positions on the *rbcL* sequences (positions 151 to 998).

**Fig 7:**
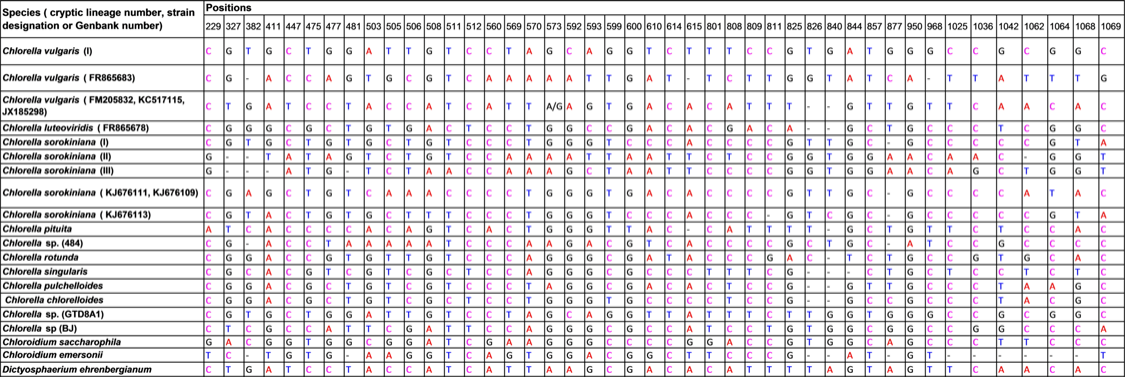
Combinations of diagnostic nucleotides for each of the 20 *Chlorella*-like taxa recovered in Fig 2. Nucleotide numbers refer to 44 selected positions on the ITS sequences (positions 229–1069).

**Fig 8:**
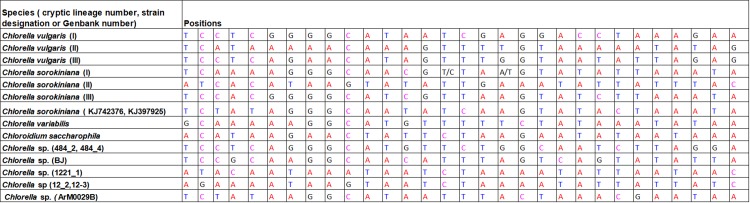
Combinations of diagnostic nucleotides for each of the 14 *Chlorella*-like taxa recovered in Fig 3. Nucleotide numbers refer to 30 selected positions on the *tufA* sequences (positions 19–673).

Based on the morphological identification, traditional barcoding, GMYC, PTP, P ID and ABGD analysis, above 31, 20 and 14 defined *Chlorella-*like clades recovered by *rbcL*, ITS and *tufA* sequences (Figs 1–3) were analyzed respectively for searching for diagnostic characters. It was shown that all the *Chlorella-*like species including the possible cryptic lineages and unknowns were clearly distinguished in the character-based DNA barcoding. In the *rbcL* gene region of 31 *Chlorella-*like taxa recovered in Fig 1, 45 character states were detected (Fig 6), in which all the 31 clades revealed a unique combination of character states at 45 nucleotide positions with more than three CAs. The possible cryptic lineages within *C*. *sorokiniana*, *D*. *ehrenbergianum* and *C*. *vulgaris*, e.g. *C*. *sorokiniana* (I),(II), *C*. *vulgaris* (I),(II) and *D*. *ehrenbergianum* (I),(II),(III) were all clearly separated with many diagnostic characters (Fig 1, Fig 6). The ITS character-based DNA barcode were shown in Fig 7, in which 20 defined *Chlorella-*like clades recovered in Fig 2 revealed a unique combination of character states, including the possible cryptic lineages *C*. *sorokiniana* (I),(II),(III) and *C*. *vulgaris* (I),(FR865683),(FM205832,KC517115,JX185298). The *tufA* character states for 14 *Chlorella-*like clades recovered in *tufA* NJ tree (Fig 3) were shown in Fig 8. At 30 nucleotide positions of the *tufA* gene region more than five CAs were revealed for each clade, also including the cryptic lineages *C*. *sorokiniana* (I),(II),(III) and *C*. *vulgaris* (I),(II),(III). In comparison with *rbcL* and ITS, *tufA* detected the most diagnostic characters in the fewest nucleotide positions. Therefore the discrimination of taxa of all clades, including cryptic and unknown taxa, could be resolved by character-based DNA barcoding.

## Discussion

This study showed that DNA barcoding based on multiple barcoding approaches was useful in species identification and cryptic species revelation of *Chlorella-*like green microalgaes. Actually, like *Chlorella*, the identification of microalgaes is often difficult due to their morphological plasticity or tiny body, which enables DNA barcoding as a powerful tool for revealing microalgae biodiversity, particularly with the combination of different analytical approaches.

### *Chlorella* primers for barcoding

DNA barcoding of plants has struggled to seek universal DNA barcodes that not only have the discriminatory power but also are easily amplified across Plantae as the COI gene chosen for animals. However, none of the available barcode loci worked well across the kingdom Plantae that includes land plants and algae [113]. For example, in molecular identification of freshwater green algae [77], the ITS1 and ITS2 regions were successfully amplified in only partial samples, which impeded the gene regions for barcoding green algae. For the barcoding of marine green macroalgae, low amplification success of *rbcL* reduced the utility of this marker as a universal barcode system [76]. In this study, the *rbcL*, 16S and *tufA* genes were successfully amplified in only a small part of *Chlorella*-like samples with the available primers. Amplification failure might be because of the primer site incompatibility in them since we have amplified the gene ITS from the same DNA templates. Thus, the *rbcL* and 16S specific primers for *Chlorella*-like strains were developed in this study based on known sequences from this study and Genbank, which turned out to be effective for amplifying and sequencing some *Chlorella-*like samples. For *tufA*, however, due to its highly variable sites and very limited available sequences, it was not possible to design the specific primers at present.

### Barcoding identification and unveiling cryptic diversity of *Chlorella-*like taxa

Identification of *Chlorella-*like organisms has long been problematic on the basis of morphological characteristics. Since most molecular studies of *Chlorella* focus on the phylogenetic analysis, the identification of *Chlorella-*like green microalgaes at species level often analyzed limited gene loci or samples. For example, for *rbcL*, a commonly used molecular marker for algae identification, prior to the initiation of this research, only six sequences of *Chlorella* species had been deposited in Genbank, and most of them were identified as unknowns. For both *Chloroidium* and *Dictyosphaerium*, only three *rbcL* sequences had been deposited in Genbank. For *tufA*, another commonly used marker, only 27 *Chlorella* sequences had been deposited in the Genbank database, and 16 of them were not identified to the species level. Moreover, the previous molecular taxonomic identification of *Chlorella-*like algae, including the use of DNA barcoding, was generally based on the phylogenetic trees or genetic distance that has often been disputed for barcoding. Therefore, the taxonomy of *Chlorella*–like species is still very complicated, and numerous cryptic species within *Chlorella-*like taxa may be still not revealed. In the present study, the barcoding analysis based on distance and character-based approaches indicate that the sequences analyzed can gave useful information into the taxonomic assessment of *Chlorella-*like strains, including the species discrimination and the revelation of unveiling complexes of possible cryptic species.

Firstly, the comprehensive barcoding analysis enabled the separation of all the specimens studied. The NJ, Bayesian and Maximum Likelihood trees produced with *rbcL*, ITS, *tufA* and 16S generally revealed congruent species delineation topologies, which revealed distinct and deeply diverged lineages. Based on the integrated analysis of GMYC, PTP, P ID and ABGD methods, the character-based barcoding of *rbcL*, ITS and *tufA* all showed clear taxa assignments that corresponded to the diverged lineages in the phylogenetic trees. Secondly, the barcoding analysis led to the revelation of potentially cryptic species in *C*. *sorokiniana*, *C*. *vulgaris* and *D*. *Ehrenbergianum*. These potentially cryptic species were all separated in all GMYC, PTP, ABGD, P ID and character-based barcoding analysis of *rbcL*, ITS and *tufA* genes. Particularly, they are clearly recovered with many diagnostic characters in character-based barcoding. In addition, to some extent, the interspecific genetic variation of *rbcL*, ITS and *tufA* among the possible cryptic lineages were higher than the intraspecific divergence (see the intra- and interspecific variation results in S3–S5 Tables). Thus, all the barcoding results revealed the potentially cryptic species complexes in *C*. *sorokiniana*, *C*. *vulgaris* and *D*. *Ehrenbergianum*. In short, the genetic data in this study indicate that there is extraordinary cryptic diversity in *Chlorella-*like taxa and further taxonomic re-evaluation of these possible cryptic species should be performed. Finally, many unknown *Chlorella*-like samples in this study did not match well with sequences published in BOLD and Genbank database. Our DNA barcoding analysis did not allow the identification of unknown specimens at the species-level. Researchers argue that barcoding is helpful in species discovery by evaluating their sequence divergence [114,115]. That is, if a matching target sequence in a barcoding database is absent the novelty of the species is generated. Thus, the unknown *Chlorella*-like specimens in this study need to be further studied. At present the DNA barcoding databases contain a limited number of reference sequences (targeted barcode) for microalgaes. More available microalgae target barcoding sequences will be greatly helpful to understand the microalgae diversity since only with an increase of accurate barcode sequences in the target databases is how DNA barcoding methods can help to produce reliable assignments of unknown species (query sequences). Also it is becoming apparent that an increased sampling may be needed to ensure the presence of a discernable barcoding gap between interspecific divergence and intraspecific variation in any given taxon and to confirm the existence of diagnostic molecular characters [50,116].

### ‘Specific barcode’ for *Chlorella-*like green microalgaes

COI was suggested as the locus that could provide recognition tags for all animals [34,35, 45]. However, COI along with other mitochondrial genes are not suitable for barcoding plantae due to their very low rates of substitution [117]. Thus, the search for plant barcodes shifted to chloroplast and nuclear genomes with high substitution rates. Despite some arguments, the most viable candidates as DNA barcode loci for plants are *rbcL* and *matK*. However, previous findings show that the *matK* or *rbcL* gene alone can not be used as a suitable universal barcode [89,91,117]. Moreover, *matK* is absent in algae. In this context, the concept of ‘specific barcode’ for plants is put forward, which involves a trade-off between single-locus barcodes and super-barcodes [91]. The ‘specific barcodes’ for different plant groups will resolve better resolution for DNA barcoding of Plantae. The *rbcL*, ITS and *tufA* have been recommend as the most promising DNA barcodes for some green algae [76,77]. In the present study, the 16S gene which has been used for identifying *C*. *vulgaris* [95] failed in discriminating most *Chlorella-*like strains, especially for the closely related species, which corresponds with the arguments that mitochondrial genes are not suitable for plant barcoding. Both of *rbcL* and ITS proved useful in distinguishing most *Chlorella-*like taxa. Yet a much higher proportion of resolution success was shown by *rbcL*, in comparison with *tufA* and ITS, including the existence of a small barcoding gap, the consistent groups among GMYC, ABGD and P ID methods, and many more diagnostic characters. Therefore, the *tufA* could be as potentially suitable ‘specific barcode’ for *Chlorella-*like taxa, which of course needs to be further compared with other gene sequences.

### Efficiency of distance and character-based DNA barcoding

Several different methods of distinguishing species have been advanced by members of the barcoding community, but which method is the best is still in debate, especially for plants [50,68,69,89,117–121]. Although the phylogenetic or distance trees in traditional barcoding approaches are informative about the genetic affinities, they are arbitrary as criterions for species identification [50,68,69]. Recently, it is proposed that incorporation of multiple lines of methodologies should be used for understanding species boundariesed framework to develop the initial species hypotheses where distinct clades are defined as those that do not share haplotypes between populations and can be identified as divergent ms, especially with the methods of GMYC, ABGD, PTP, P IN and CAOS [109, 122–124]. It has also been proposed that an optimal path to understand species boundaries is starting with a tree or distance-baonophyletic population clusters [109]. Then the character-based approach is employed to confirm the initial identification. Our study represents one of the first efforts to test the congruence of barcoding results from multiple delimitation methods.

For traditional barcoding, generally, the NJ, Bayesian and Maximum Likelihood analysis recovered consistent topology for each gene of *rbcL*, ITS, 16S and *tufA*. However, due to the shortcoming of tree-based species identification [55, 60] the phylogenetic trees are more likely to be used initially to identify putative independently-evolving lineages. The intra and inter-specific distance of traditinal barcoding was also analyzed in this study. To some degree, the distance method was helpful in species discrimination. For example, as a whole, the interspecific variation of *rbcL*, ITS and *tufA* sequences among the potentially cryptic species complexes in *C*. *sorokiniana*, *C*. *vulgaris* and *D*. *Ehrenbergianum* was higher than the intraspecific variation. Nevertheless, for all of *rbcL*, ITS and *tufA* sequences, although the interspecific genetic variation was generally higher than the intraspecific genetic varation, there was no apparent barccoding gap between them (seen S3–S5 Tables). That is, the minimum interspecific distance is smaller than the maximum intraspecific distance, which contradicts the criterion of species identification with sequences distance [89].

The resolution produce by GMYC, PTP, P ID, ABGD and character-based barcoding methods were variable in each of *rbcL*, ITS and *tufA* genes. In *rbcL* analysis, the groups recovered by GMYC, ABGD and PTP methods were consistent while the groups recovered by P ID and character methods were consistent. In ITS analysis, the groups produced by GMYC, ABGD and PTP methods were all different from each other while P ID and character methods produced same groups. The best resolution for species differentiation appeared in *tufA* analysis where GMYC, PTP, ABGD and character-based approaches produced consistent groups while the PTP method over-split the taxa. Similar to previous studies [122, 125–129], GMYC typically generates more OTUs (operational taxonomic units) than other approaches for *rbcL* sequences and errors in the ultrametric gene tree will influence final results. The PTP, however, generate more OTUs than other methods in both ITS and *tufA* genes. Generally, the P ID and character-based methods produced consistent groups in all *rbcL*, ITS and *tufA* genes.

Based on the integrated analysis of traditional barcoding, GMYC, ABGD, PTP and P ID methods, the putative species recovered were confirmed by character-based barcoding. The character-based DNA barcoding showed more advantages, particularly for revealing the possible cryptic lineages. For example, as expected, the character-based analysis generated relatively congruent results in *rbcL*, ITS and *tufA* genes, and most taxonomic groups analyzed by *rbcL*, ITS, 16S and *tufA* genes, including the potentially cryptic species, possessed unique simple identifying character states in character-based barcoding. Some species that could not be discriminated with traditional barcoding, GMYC, PTP or ABGD methods could be detected by character-based method, e.g. *Chlorella vulgaris* (I) and *Chlorella sorokiniana* (I) in *rbcL* barcoding analysis, and *Chlorella sorokiniana* (I) in ITS barcoding analysis (Figs 1–3). In addition, if one species is represented with only a single individual or not all closely related species are sampled it is not possible to determine the correct intra- and interspecific divergences, which may hinder the presence of a discernable barcoding gap. Nevertheless, a single individual can be still assigned to a distinct clade in character-based DNA barcode. In this study, quite a few *Chlorella-*like taxa represented with only a single individual were clearly distinguished with unique combination of character attributes, especially for the unknowns. This is particularly useful for flagging hidden new species. Thus, a character-based discrimination criterion can maximize the success rate of molecular identification in *Chlorella*-like organism, which can resolve cases that the coalescent and distance-based barcoding does not. It may be an optimal option to first combine multiple barcoding approaches to test primary species hypotheses species and then confirm the taxonomic assignments by the character-based method. Future DNA barcoding of comprehensive *Chlorella-*like green microalgaes with character-based analysis may move towards a better understanding of this morphologically complex microalgaes.

## Conclusion

This study indicates that the combination of *rbcL*, ITS and *tufA* sequence data analyzed by combination of GMYC, ABGD, PTP, P ID and character-based barcoding is very useful to discriminate the *Chlorella*-like samples and reveal the complexes of potentially cryptic species that merit further study. The resolution produced by GMYC, PTP, P ID, ABGD and character-based barcoding methods were variable in each of *rbcL*, ITS and *tufA* genes. The *tufA* produced consistent groups among GMYC, ABGD, P ID and character-based methods and also offered many more diagnostic characters than *rbcL* and ITS. The *tufA* region thus could be as potentially suitable ‘specific barcode’ for *Chlorella-*like taxa. On the other hand, all the character analysis of *rbcL*, ITS and *tufA* sequence could clearly distinguish all taxonomic groups, including the potentially cryptic lineages, with many character attributes. In comparison with other barcoding methods, the character-based discrimination criterion can maximize the success rate of molecular identification in *Chlorella*-like organisms, which can resolve cases that the distance and coalescent-based criterion does not. The character-based barcoding could be used as an attractive complement to coalescent and distance-based barcoding. It could be an optimal option to first combine multiple barcoding approaches to test primary species hypotheses species and then confirm the taxonomic assignments by the character-based method. Further DNA barcoding of comprehensive *Chlorella-*like green microalgaes with character-based analysis may move towards a better understanding of this morphologically complex microalgaes.

## Supporting Information

S1 Fig Maximum Likelihood tree for the *rbcL* gene. (TIF)

S2 Fig Maximum Likelihood tree for the ITS gene. (TIF)

S3 Fig Maximum Likelihood tree for the *tufA* gene. (TIF)

S4 Fig Bayesian phylogenetic tree for the 16S gene. Posterior probabilities and NJ bootstrap values were included.
(TIF)

S5 Fig Maximum Likelihood tree for the 16S gene. (TIF)

S6 Fig Automatic partition of tellinaceans based on *rbcL* gene. The number of groups inside the partition (initial and recursive) of each given prior intraspecific divergence value were reported.
(JPG)

S7 Fig Automatic partition of tellinaceans based on ITS gene. The number of groups inside the partition (initial and recursive) of each given prior intraspecific divergence value were reported.
(JPG)

S8 Fig Automatic partition of tellinaceans based on *tufA* gene. The number of groups inside the partition (initial and recursive) of each given prior intraspecific divergence value were reported.
(JPG)

S9 Fig GMYC resulution of *rbcL* genes. The red vertical line in the tree was the threshold point obtained from the GMYC model.
(TIF)

S10 Fig GMYC resulution of ITS genes. The red vertical line in the tree was the threshold point obtained from the GMYC model.
(TIF)

S11 Fig GMYC resulution of *tufA* genes. The red vertical line in the tree was the threshold point obtained from the GMYC model.
(TIF)

S1 Table List of specimens with the classification, collection details, and voucher numbers. (DOC)

S2 Table Primer sequences and annealing temperatures used to amplify the different regions. (DOC)

S3 Table The mean interspecific divergencesof *rbcL* sequences for *Chlorella-*like taxa. (DOC)

S4 Table Themean interspecific divergences of ITS sequences for *Chlorella-*like taxa. (DOC)

S5 Table The mean interspecific divergencesof *tufA* sequences for *Chlorella-*like taxa. (DOC)

S6 Table Species Delimitation Results of PID for *rbcL*. The species number (clade) corresponds to the P ID clades in Fig 1.
(XLSX)

S7 Table Species Delimitation Results of PID for ITS. The species number (clade) corresponds to the P ID clades in Fig 2.
(XLSX)

S8 Table Species Delimitation Results of PID for *tufA*. The species number (clade) corresponds to the P ID clades in Fig 3.
(XLSX)
