# Supplementary figures and images for: Combining and Comparing Coalescent, Distance and Character-Based Approaches for Barcoding Microalgaes: A Test with Chlorella-Like Species (Chlorophyta)
Source: PLoS One. 2016 Apr 19;11(4):e0153833. doi: 10.1371/journal.pone.0153833 (PMC4841637; doi:10.1371/journal.pone.0153833)

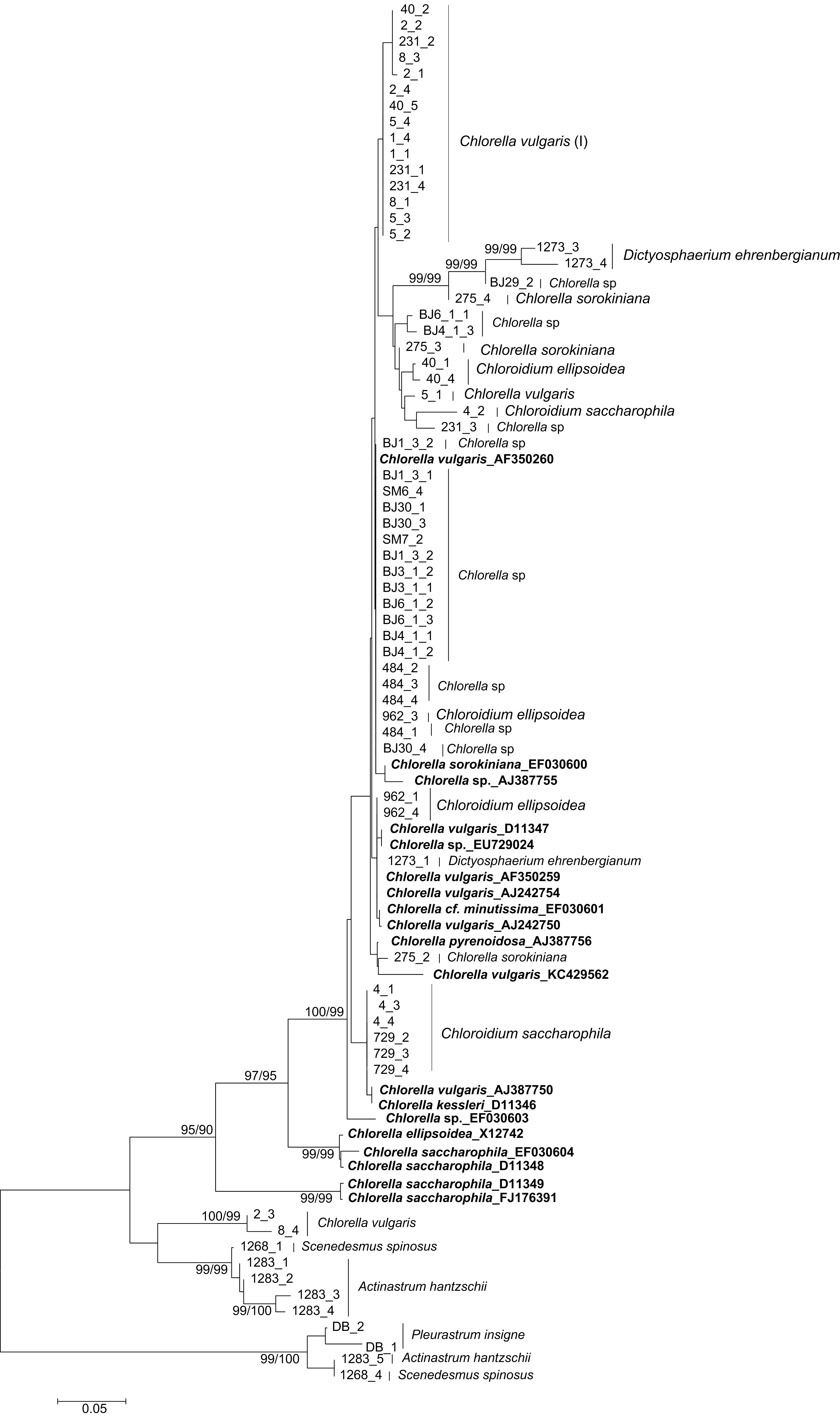

Supplement: S4 Fig — Posterior probabilities and NJ bootstrap values were included. (TIF) [file pone.0153833.s004.tif]

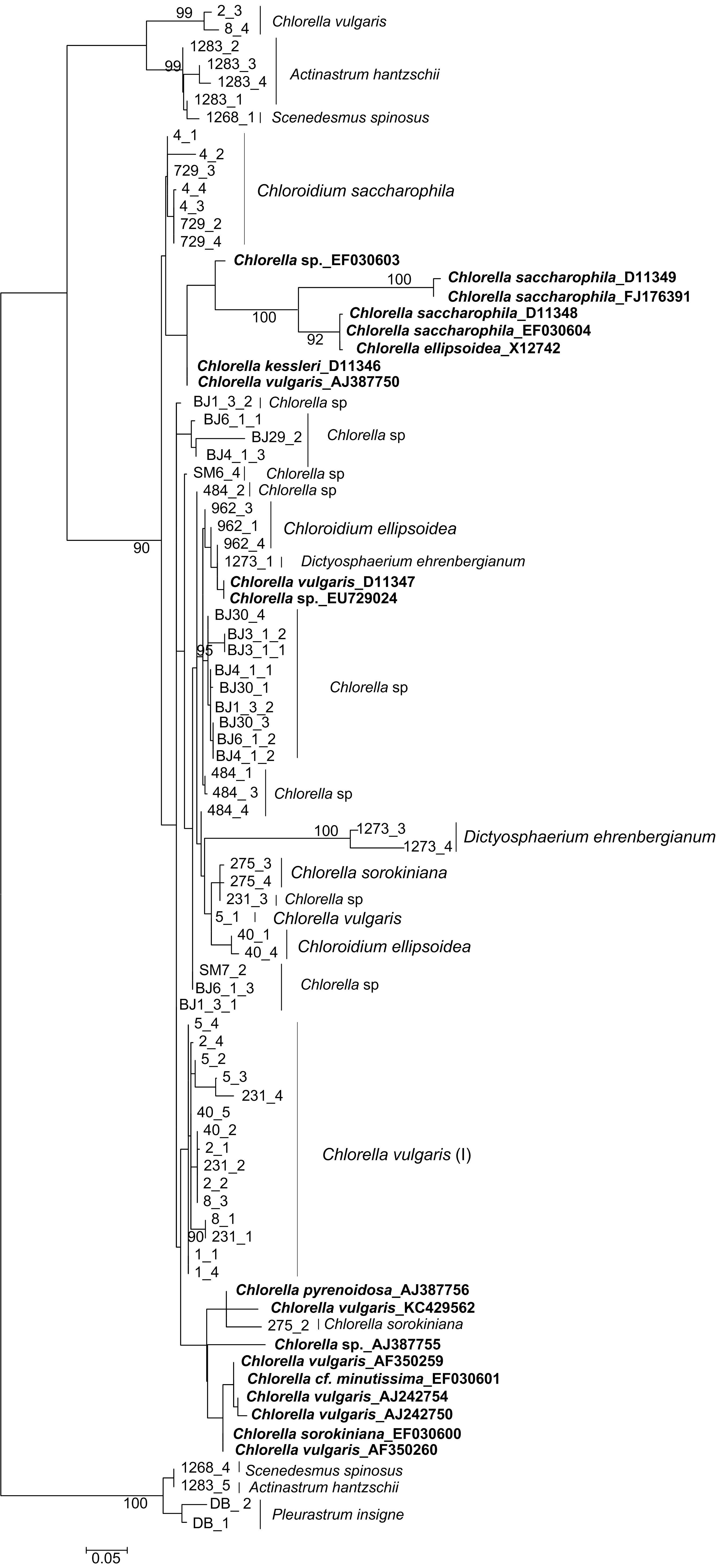

Supplement: S5 Fig — (TIF) [file pone.0153833.s005.tif]

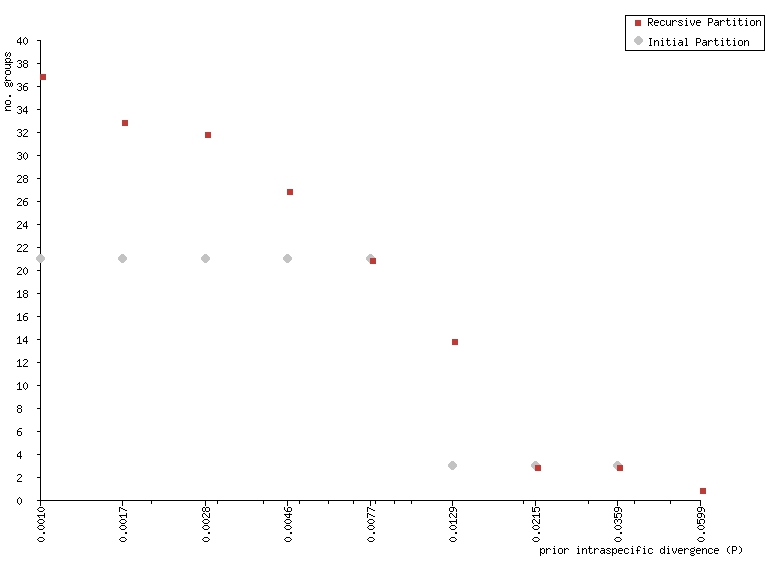

Supplement: S6 Fig — The number of groups inside the partition (initial and recursive) of each given prior intraspecific divergence value were reported. (JPG) [file pone.0153833.s006.jpg]

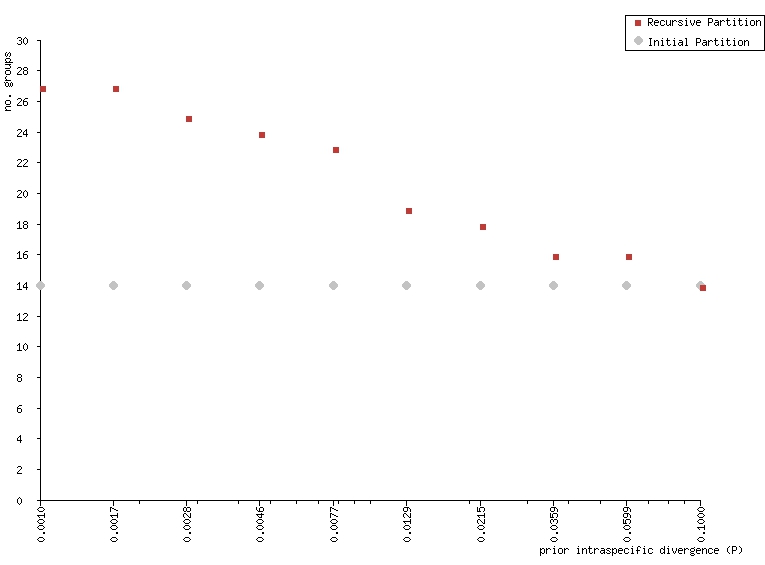

Supplement: S7 Fig — The number of groups inside the partition (initial and recursive) of each given prior intraspecific divergence value were reported. (JPG) [file pone.0153833.s007.jpg]

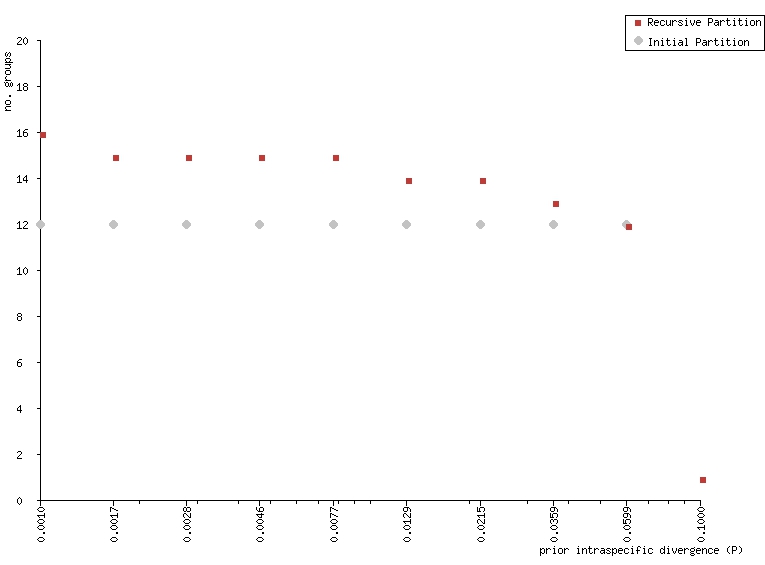

Supplement: S8 Fig — The number of groups inside the partition (initial and recursive) of each given prior intraspecific divergence value were reported. (JPG) [file pone.0153833.s008.jpg]

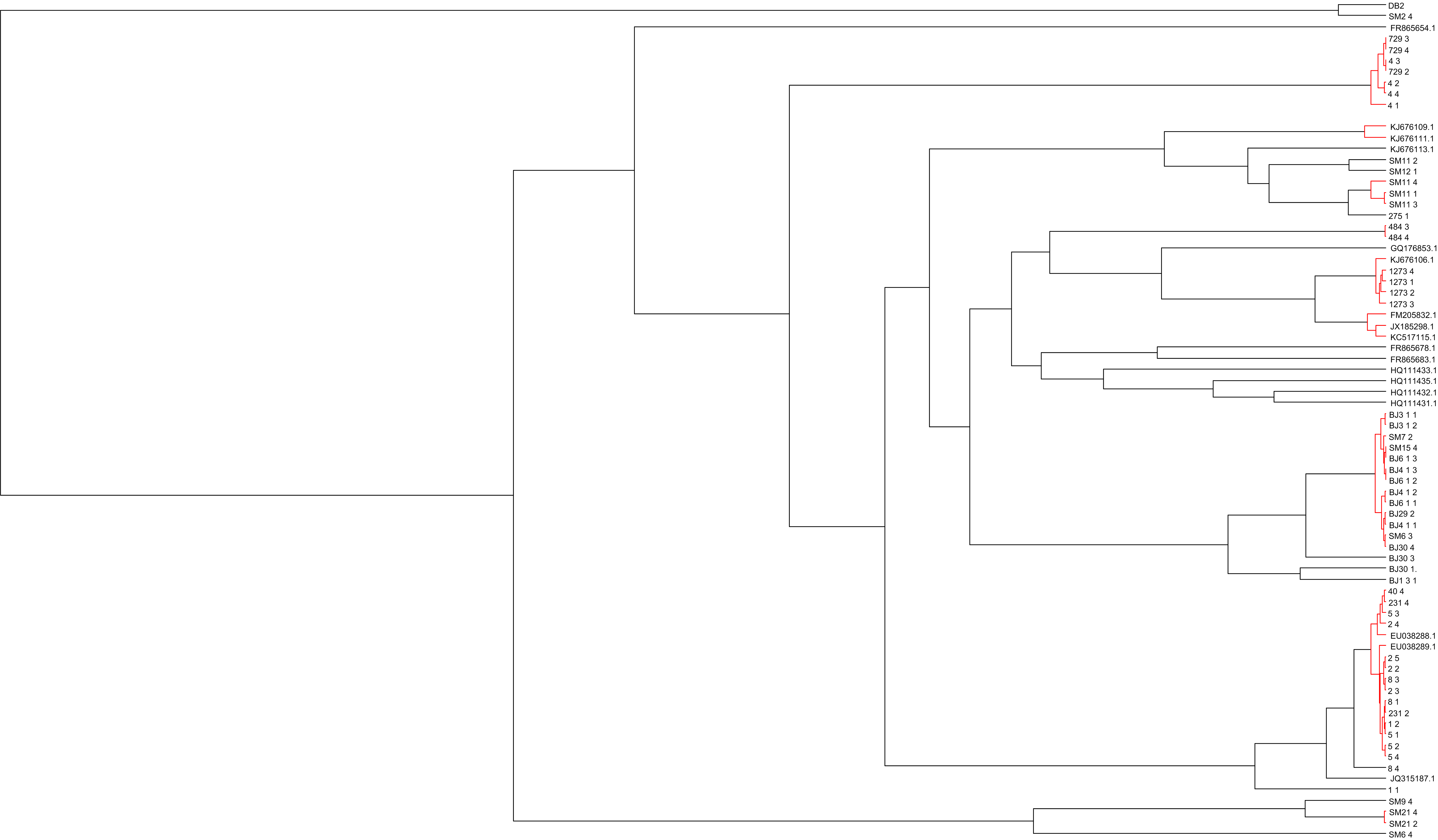

Supplement: S10 Fig — The red vertical line in the tree was the threshold point obtained from the GMYC model. (TIF) [file pone.0153833.s010.tif]

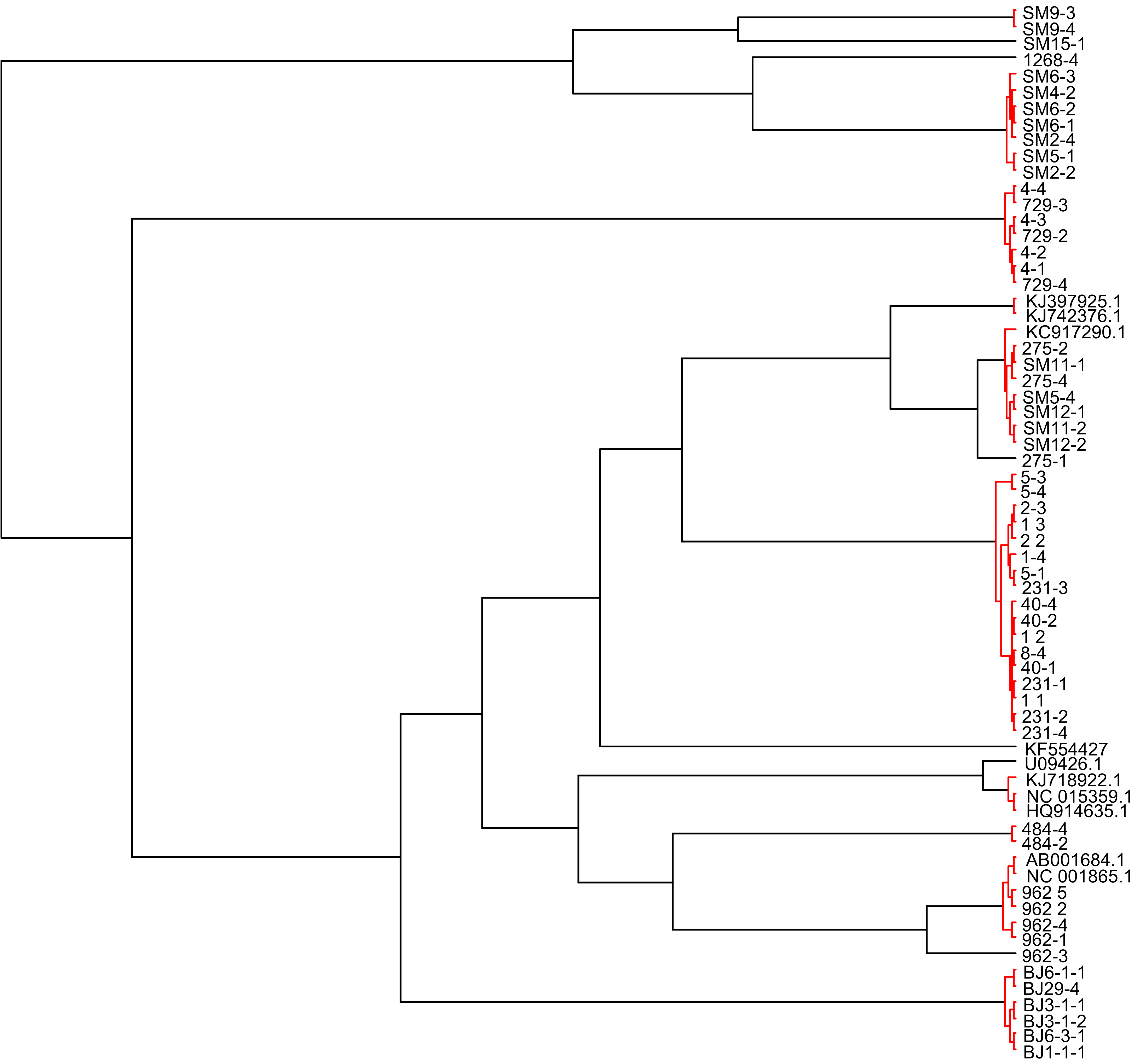

Supplement: S11 Fig — The red vertical line in the tree was the threshold point obtained from the GMYC model. (TIF) [file pone.0153833.s011.tif]
