# Supplementary material for: Combining and Comparing Coalescent, Distance and Character-Based Approaches for Barcoding Microalgaes: A Test with Chlorella-Like Species (Chlorophyta)
Source: PLoS One. 2016 Apr 19;11(4):e0153833. doi: 10.1371/journal.pone.0153833 (PMC4841637; doi:10.1371/journal.pone.0153833)
Supplement: S1 Table — (DOC) [file pone.0153833.s012.doc]

**S1** **Table** List of specimens with the classification, collection details, and voucher numbers. GenBank accession numbers are also given, some of which in bold were previously published.

|  |  | |  | | **GenBank accession number** | | | | | | | |  |
| --- | --- | --- | --- | --- | --- | --- | --- | --- | --- | --- | --- | --- | --- |
| **Strain designation** | **Collection locality** | | **Species** | | ***rbcL*** | | **ITS** | | **16S** | | ***tufA*** | |  |
| ***Chlorella*** |  | |  | |  | |  | |  | |  | |  |
| 1_1 | Sea water in Qingdao, Shandong, China | | *C. vulgaris* | | KM514869 | | KM514815 | | KM514785 | | KR154285 | |  |
| 1_2 | Sea water in Qingdao, Shandong, China | | *C. vulgaris* | |  | | KM514847 | |  | | KR154287 | |  |
| 1_4 | Charles University in Prague | | *C. vulgaris* | |  | |  | | KM514786 | | KR154257 | |  |
| 2--1 | Lake Taihu, Jiangsu, China | | *C. vulgaris* | |  | | KM514849 | | KM514800 | |  | |  |
| 2--2 | Lake Taihu, Jiangsu, China | | *C. vulgaris* | | KM514871 | | KM514848 | | KM514799 | | KR154240 | |  |
| 2--3 | Shihezi, Xinjiang, China | | *C. vulgaris* | |  | | KM514816 | | KM514744 | | KR154258 | |  |
| 2--4 | Lake Xuanwuhu, Jiangsu, China | | *C. vulgaris* | |  | | KM514841 | | KM514797 | |  | |  |
| 5--1 | Pond at state New York, USA | | *C. vulgaris* | |  | | KM514825 | | KM514758 | | KR154261 | |  |
| 5--2 | Austin, Texas, Waller Creek at University Campus, USA, | | *C. vulgaris* | | KM514874 | | KM514842 | | KM514759 | |  | |  |
| 5--3 | Lake Donghu, Wuhan, China | | *C. vulgaris* | | KM514875 | | KM514826 | | KM514760 | | KR154262 | |  |
| 5--4 | Soil from Shihezi, Xinjiang, China | | *C. vulgaris* | | KM514876 | | KM514843 | | KM514787 | | KR154263 | |  |
| 8--1 | Fuzhou, Fujian, China | | *C. vulgaris* | |  | | KM514844 | | KM514761 | |  | |  |
| 8--3 | Lake Xuanwuhu, Jiangsu, China | | *C. vulgaris* | | KM514877 | | KM514828 | | KM514762 | |  | |  |
| 8--4 | Lake Taihu, Jiangsu, China | | *C. vulgaris* | | KM514878 | | KM514829 | | KM514763 | | KR154265 | |  |
| 40--1 | Lake Hulunbeier, Neimeng, China | | *C. vulgaris* | | KM514879 | |  | | KM514764 | | KR154266 | |  |
| 40--2 | Lake Hulunbeier, Neimeng, China | | *C. vulgaris* | |  | |  | | KM514788 | | KR154267 | |  |
| 40--3 | Lake Donghu, Wuhan, China | | *C. vulgaris* | |  | |  | |  | |  | |  |
| 40--4 | Lake Donghu, Wuhan, China | | *C. vulgaris* | | KM514880 | | KM514832 | | KM514765 | | KR154268 | |  |
| 40--5 | Lake Donghu, Wuhan, China | | *C. vulgaris* | | KM514881 | |  | | KM514789 | |  | |  |
| 962--1 | Seawater in Qingdao, China | | *C. vulgaris* | | KM514894 | |  | | KM514776 | | KR154281 | |  |
| 962--2 | Seawater in Lianyungang, China | | *C. vulgaris* | | KM514895 | |  | |  | | KR154236 | |  |
| 962--3 | Seawater in Qingdao, China | | *C. vulgaris* | | KM514896 | |  | | KM514777 | | KR154282 | |  |
| 962--4 | Seawater in Qingdao, China | | *C. vulgaris* | | KM514897 | |  | | KM514793 | | KR154283 | |  |
| 962--5 | Seawater in Lianyungang, China | | *C. vulgaris* | |  | |  | |  | | KR154237 | |  |
| 231--1 | Pond, Wuhan,China | | *C. vulgaris* | | KM514882 | |  | | KM514768 | | KR154246 | |  |
| 231--2 | Pond, Wuhan,China | | *C. vulgaris* | | KM514883 | | KM514833 | | KM514769 | | KR154247 | |  |
| 231--3 | Seawater in Lianyungang, Jiangsu, China | | *C. vulgaris* | | KM514885 | |  | | KM514766 | | KR154248 | |  |
| 231--4 | Lake Zixia,Nanjing, Jiangsu | | *C. vulgaris* | | KM514886 | | KM514834 | | KM514767 | | KR154249 | |  |
| F-5 |  | | *C. vulgaris* | | **EU038282** | |  | |  | |  | |  |
| F-9 |  | | *C. vulgaris* | | **EU038283** | |  | |  | |  | |  |
|  |  | | *C. vulgaris* | | **AB240145** | |  | |  | |  | |  |
|  |  | | *C. vulgaris* | | **KC810313** | |  | |  | |  | |  |
| LU2 |  | | *C. vulgaris* | | **JQ717305** | |  | |  | |  | |  |
| 820 |  | | *C. vulgaris* | | **EU038284** | |  | |  | |  | |  |
| IAM C-210 |  | | *C. vulgaris* | |  | |  | | **AJ387756** | |  | |  |
|  |  | | *C. vulgaris* | |  | | **EU038289** | |  | |  | |  |
|  |  | | *C. vulgaris* | |  | | **EU038288** | |  | |  | |  |
| Cvq |  | | *C. vulgaris* | | **EU038286** | |  | |  | |  | |  |
|  |  | | *C. vulgaris* | | **AB260909** | |  | |  | |  | |  |
|  |  | | *C. vulgaris* | | **KC810317** | |  | |  | |  | |  |
|  |  | | *C. vulgaris* | | **JQ415918** | |  | |  | |  | |  |
| LU18 |  | | *C. vulgaris* | | **JQ415915** | |  | |  | |  | |  |
|  |  | | *C. vulgaris* | | **AF499684** | |  | |  | |  | |  |
| CCAP 254/5 |  | | *C. vulgaris* | | **EF589154** | |  | |  | |  | |  |
| SAG 211-11b |  | | *C. vulgaris* | |  | | **FM205832** | |  | |  | |  |
| D2 |  | | *C. vulgaris* | |  | | **JX185298** | |  | |  | |  |
| CSA-53 |  | | *C. vulgaris* | |  | | **KC517115** | |  | |  | |  |
| CCAP 211/79 |  | | *C. vulgaris* | |  | | **FR865683** | |  | |  | |  |
|  |  | | *C. vulgaris* | |  | |  | | **AF350260** | |  | |  |
|  |  | | *C. vulgaris* | |  | |  | | **AF350259** | |  | |  |
| DGGE |  | | *C. vulgaris* | |  | |  | | **KC429562** | |  | |  |
| IAM C-208 |  | | *C. vulgaris* | |  | |  | | **AJ387750** | |  | |  |
| NIES 227 |  | | *C. vulgaris* | |  | |  | | **AJ242754** | |  | |  |
| CCAP 211/19 |  | | *C. vulgaris* | |  | |  | | **AJ242750** | |  | |  |
| 2111e |  | | *C. vulgaris* | |  | |  | | **D11347** | |  | |  |
| 21111h |  | | *C. vulgaris* | |  | |  | | **D11346** | |  | |  |
| C-27 |  | | *C. vulgaris* | |  | |  | |  | | **AB001684** | |  |
|  |  | | *C. vulgaris* | |  | |  | |  | | **NC001865** | |  |
|  |  | |  | |  | |  | |  | |  | |  |
| 1_3 | Shihezi, Xinjiang, China | | *C. luteoviridis* | | KM514870 | |  | |  | | KR154286 | |  |
| UTEX 21 |  | | *C. luteoviridis* | | **EF113428** | |  | |  | |  | |  |
|  |  | | *C. luteoviridis* | |  | | **FR865678** | |  | |  | |  |
|  |  | |  | |  | |  | |  | |  | |  |
| 275-1 | Austin, Texas, Waller Creek at University Campus, USA, 2000 | | *C. sorokiniana* | | KM514887 | | KM514835 | |  | | KR154269 | |  |
| 275-2 | Lake Xuanwuhu, Nanjing, China | | *C. sorokiniana* | | KM514888 | |  | | KM514801 | | KR154270 | |  |
| 275-3 | Austin, Texas, Waller Creek at University Campus, USA, 1987 | | *C. sorokiniana* | |  | |  | | KM514790 | |  | |  |
| 275-4 | Austin, Texas, Waller Creek at University Campus, USA, 1990 | | *C. sorokiniana* | | KM514865 | |  | | KM514791 | | KR154271 | |  |
| SM11-1 | Soil from Shihezi, Xinjiang, China | | *C. sorokiniana* | |  | | KM514855 | |  | | KR154291 | |  |
| SM11-2 | Soil from Shihezi, Xinjiang, China | | *C. sorokiniana* | |  | | KM514856 | |  | | KR154253 | |  |
| SM11-3 | Soil from Shihezi, Xinjiang, China | | *C. sorokiniana* | | KM514907 | | KM514857 | |  | |  | |  |
| SM11-4 | Soil from Shihezi, Xinjiang, China | | *C. sorokiniana* | |  | | KM514858 | |  | |  | |  |
| SM12-1 | Soil from Shihezi, Xinjiang, China | | *C. sorokiniana* | |  | | KM514859 | |  | | KR154254 | |  |
| SM12-2 | Soil from Shihezi, Xinjiang, China | | *C. sorokiniana* | | KM514884 | |  | |  | | KR154255 | |  |
| SM15-3 | Lake Hulunbeier, Neimeng, China | | *C. sorokiniana* | | KM514908 | |  | |  | |  | |  |
| SM15-4 | Lake Hulunbeier, Neimeng, China | | *C. sorokiniana* | | KM514910 | | KM514813 | |  | | KR154276 | |  |
| SM21-4 | Lake Hulunbeier, Neimeng, China | | *C. sorokiniana* | | KM514903 | | KM514831 | |  | | KR154289 | |  |
| SM21-2 | Lake Hulunbeier, Neimeng, China | | *C. sorokiniana* | | KM514901 | | KM514830 | |  | | KR154238 | |  |
| SM21-3 | Lake Hulunbeier, Neimeng, China | | *C. sorokiniana* | | KM514902 | |  | |  | |  | |  |
| SM18-1 | Seawater in Qingdao, China | | *C. sorokiniana* | | KM514916 | |  | |  | | KR154277 | |  |
| SM18-2 | Lake Taihu, Jiangsu, China | | *C. sorokiniana* | |  | |  | |  | | KR154278 | |  |
| SM18-3 | Lake Taihu, Jiangsu, China | | *C. sorokiniana* | | KM514917 | |  | |  | | KR154279 | |  |
| SM9-2 | Lake Taihu, Jiangsu, | | *C. sorokiniana* | | KM514904 | |  | |  | | KR154275 | |  |
| SM9-4 | Seawater in Qingdao, China | | *C. sorokiniana* | |  | | KM514851 | |  | |  | |  |
| SM10-3 | Seawater in Qingdao, China | | *C. sorokiniana* | | KM514905 | |  | |  | | KR154280 | |  |
| SM10-4 | Seawater in Qingdao, China | | *C. sorokiniana* | | KM514906 | |  | |  | |  | |  |
| UTEX 246 |  | | *C. sorokiniana* | | **EF113429** | |  | |  | |  | |  |
|  |  | | *C. sorokiniana* | | **KC810315** | |  | |  | |  | |  |
| LU5 |  | | *C. sorokiniana* | | **JQ415926** | |  | |  | |  | |  |
| LU10 |  | | *C. sorokiniana* | | **JQ415922** | |  | |  | |  | |  |
| LU14 |  | | *C. sorokiniana* | | **JQ415921** | |  | |  | |  | |  |
|  |  | | *C. sorokiniana* | | **HM101339** | |  | |  | |  | |  |
|  |  | | *C. sorokiniana* | |  | | **KJ676109** | |  | |  | |  |
|  |  | | *C. sorokiniana* | |  | | **KJ676111** | |  | |  | |  |
|  |  | | *C. sorokiniana* | |  | | **KJ676113** | |  | |  | |  |
| SM6-4 | Lake Taihu, Jiangsu, | | *C. sorokiniana* | |  | | KM514806 | | KM514740 | | KR154284 | |  |
|  |  | | *C. sorokiniana* | |  | |  | | **EF030600** | |  | |  |
| 1230 |  | | *C. sorokiniana* | |  | |  | |  | | **KJ742376** | |  |
|  |  | | *C. sorokiniana* | |  | |  | |  | | **KJ397925** | |  |
| 2009100806 |  | | *C. sorokiniana* | |  | |  | |  | | **KC917290** | |  |
| IFRPD 1018 |  | | *C. sorokiniana* | | **AB260911** | |  | |  | |  | |  |
|  |  | |  | |  | |  | |  | |  | |  |
| Syngen 2-3 |  | | *C. variabilis* | | **AB260904** | |  | |  | |  | |  |
| NC64A |  | | *C. variabilis* | | **AB260903** | |  | |  | |  | |  |
|  |  | | *C. variabilis* | | **AB260902** | |  | |  | |  | |  |
|  |  | | *C. variabilis* | | **AB260901** | |  | |  | |  | |  |
| n1a |  | | *C. variabilis* | |  | |  | |  | | **U09426** | |  |
|  |  | | *C. variabilis* | |  | |  | |  | | **NC015359** | |  |
| NC64A |  | | *C. variabilis* | |  | |  | |  | | **KJ718922** | |  |
|  |  | | *C. variabilis* | |  | |  | |  | | **HQ914635** | |  |
|  |  | | *C. minutissima* | | **KC810312** | |  | |  | |  | |  |
|  |  | | *C. minutissima* | |  | |  | | **EF030601** | |  | |  |
| CB 2008/50 |  | | *C. pulchelloides* | |  | | **HQ111431** | |  | |  | |  |
| KR 2007/5 |  | | *C. rotunda* | |  | | **HQ111433** | |  | |  | |  |
|  |  | | *C. chlorelloides* | |  | | **HQ111432** | |  | |  | |  |
| UTEX1805 |  | | *C. parva* | |  | | **KJ676106** | |  | |  | |  |
|  |  | | *C. singularis* | |  | | **HQ111435** | |  | |  | |  |
|  |  | | *C. singularis* | | **KC810314** | |  | |  | |  | |  |
| ACOI 311 |  | | *C. pituita* | |  | | **GQ176853** | |  | |  | |  |
|  |  | | *C. emersonii* | |  | | **FR865654** | |  | |  | |  |
|  |  | |  | |  | |  | |  | |  | |  |
| 484-1 | Lake Mochou, Nanjing, China | | *C*. sp. | | KM514889 | |  | | KM514792 | |  | |  |
| 484-2 | Lake Mochou, Nanjing, China | | *C*. sp. | | KM514890 | |  | | KM514770 | | KR154250 | |  |
| 484-3 | Lake Taihu, Jiangsu, China | | *C*. sp. | | KM514866 | | KM514836 | | KM514771 | |  | |  |
| 484-4 | Lake Taihu, Jiangsu, China | | *C*. sp. | |  | | KM514837 | | KM514772 | | KR154251 | |  |
|  |  | | *C*. sp. | |  | |  | |  | |  | |  |
| BJ1-3-2 | Glacier in Arctic pole | | *C*. sp. | | KM514911 | |  | | KM514743 | |  | |  |
| BJ1-3-1 | Glacier in Arctic pole | | *C*. sp. | |  | | KM514852 | | KM514742 | | KR154239 | |  |
| BJ1-3-3 | Glacier in Arctic pole | | *C*. sp. | |  | |  | | KM514803 | |  | |  |
| BJ3-1-2 | Glacier in Arctic pole | | *C*. sp. | | KM514912 | | KM514854 | | KM514745 | | KR154259 | |  |
| BJ3-1-1 | Glacier in Arctic pole | | *C*. sp. | |  | | KM514853 | | KM514746 | | KR154241 | |  |
| BJ4-1-1 | Glacier in Arctic pole | | *C*. sp. | | KM514909 | | KM514860 | | KM514756 | |  | |  |
| BJ4-1-2 | Glacier in Arctic pole | | *C*. sp. | | KM514913 | | KM514820 | | KM514757 | |  | |  |
| BJ4-1-3 | Glacier in Arctic pole | | *C*. sp. | |  | | KM514823 | | KM514750 | |  | |  |
| BJ6-1-1 | Glacier in Arctic pole | | *C*. sp. | | KM514914 | | KM514824 | | KM514749 | | KR154264 | |  |
| BJ6-1-2 | Glacier in Arctic pole | | *C*. sp. | | KM514915 | | KM514822 | | KM514804 | | KR154245 | |  |
| BJ6-1-3 | Glacier in Arctic pole | | *C*. sp. | |  | | KM514827 | | KM514754 | |  | |  |
| BJ29-2 | Glacier in Arctic pole | | *C*. sp. | |  | | KM514807 | | KM514739 | | KR154288 | |  |
| BJ30-3 | Glacier in Arctic pole | | *C*. sp. | |  | | KM514809 | | KM514795 | |  | |  |
| BJ30-4 | Glacier in Arctic pole | | *C*. sp. | |  | | KM514808 | | KM514753 | |  | |  |
| BJ30-1 | Glacier in Arctic pole | | *C*. sp. | |  | | KM514805 | | KM514738 | |  | |  |
| SM15-4 | Soil from Shihezi, Xinjiang, China | | *C*. sp. | |  | | KM514813 | |  | |  | |  |
| SM7-2 | Soil from Shihezi, Xinjiang, China | | *C*. sp. | |  | | KM514814 | | KM514741 | |  | |  |
| 1221-1 | Reservoir，Lushuihu, Hubei, China | | *C*. sp. | | KM514867 | |  | |  | | KR154256 | |  |
| 12--2 | Huangshi, Hubei, China, 1960 | | *C*. sp. | | KM514863 | |  | |  | | KR154290 | |  |
| LU8 |  | | *C*. sp. | | **JQ415924** | |  | |  | |  | |  |
| LUCC 020 |  | | *C*. sp. | | **KC810316** | |  | |  | |  | |  |
| LU28 |  | | *C*. sp. | | **JQ713930** | |  | |  | |  | |  |
|  |  | | *C*. sp. | |  | |  | |  | |  | |  |
| L9 |  | | *C*. sp. | | **AB713417** | |  | |  | |  | |  |
| M9 |  | | *C*. sp. | | **AB713419** | |  | |  | |  | |  |
| J8 |  | | *C*. sp. | | **AB713416** | |  | |  | |  | |  |
| KMMCC 868 |  | | *C*. sp. | | **JQ315474** | |  | |  | |  | |  |
| GTD8A1 |  | | *C*. sp. | |  | | **JQ315187** | |  | |  | |  |
|  |  | | *C*. sp. | |  | |  | | **EU729024** | |  | |  |
|  |  | | *C*. sp. | |  | |  | | **AJ387755** | |  | |  |
| HvMH |  | | *C*. sp. | |  | |  | | **EF030603** | |  | |  |
| ArM0029B |  | | *C*. sp. | |  | |  | |  | | **KF554427** | |  |
|  |  | |  | |  | |  | |  | |  | |  |
| ***Chloroidium*** |  | |  | |  | |  | |  | |  | |  |
| 4--1 | Charles University in Prague | | *C. saccharophila* | | KM514872 | | KM514818 | | KM514751 | | KR154242 | |  |
| 4--2 | Lake Hulunbeier, Neimeng, China | | *C. saccharophila* | |  | | KM514819 | | KM514748 | | KR154243 | |  |
| 4--3 | Lake Zixia,Nanjing, Jiangsu | | *C. saccharophila* | |  | | KM514771 | | KM514752 | | KR154244 | |  |
| 4--4 | Charles University in Prague | | *C. saccharophila* | | KM514873 | | KM514821 | | KM514755 | | KR154260 | |  |
|  |  | | *C. saccharophila* | |  | |  | | **EF030604** | |  | |  |
| 211-1d |  | | *C. saccharophila* | |  | |  | | **D11349** | |  | |  |
| 3.8 |  | | *C. saccharophila* | |  | |  | | **D11348** | |  | |  |
| UTEX 2911 |  | | *C. saccharophila* | |  | |  | | **FJ176391** | |  | |  |
| 729--1 | University of Texas, 1987 | | *C. saccharophila* | | KM514891 | |  | |  | |  | |  |
| 729--2 | University of Texas, 1987 | | *C. saccharophila* | | KM514892 | | KM514838 | | KM514773 | | KR154272 | |  |
| 729--3 | Pond at state New York, USA | | *C. saccharophila* | |  | | KM514845 | | KM514774 | | KR154273 | |  |
| 729--4 | Pond at state New York, USA | | *C. saccharophila* | | KM514893 | | KM514839 | | KM514775 | | KR154274 | |  |
| Ce |  | | *C. ellipsoidea* | | **EU038287** | |  | |  | |  | |  |
| CCAP 211/33 |  | | *C. ellipsoidea* | | **EF589156** | |  | |  | |  | |  |
|  |  | | *C. ellipsoidea* | |  | |  | | **X12742** | |  | |  |
|  |  | |  | |  | |  | |  | |  | |  |
| ***Dictyosphaerium*** | |  | |  | |  | |  | |  | |  | |
| 1273-1 | Lake Taihu, China | | *D. ehrenbergianum* | | KM514898 | | KM514850 | | KM514798 | |  | |  |
| 1273-3 | Lake Hulunbeier, Neimeng, China | | *D. ehrenbergianum* | | KM514864 | | KM514840 | | KM514779 | |  | |  |
| 1273-2 | Lake Taihu, China | | *D. ehrenbergianum* | | KM514868 | |  | |  | |  | |  |
| 1273-4 | Lake Hulunbeier, Neimeng, China | | *D. ehrenbergianum* | | KM514899 | | KM514846 | | KM514780 | |  | |  |
|  |  | |  | |  | |  | |  | |  | |  |
| ***Actinastrum*** |  | |  | |  | |  | |  | |  | |  |
| 1283-1 | Pond Guanqiao, Jingzhou, China | | *A. hantzschii* | |  | |  | | KM514783 | |  | |  |
| 1283-2 | Lake, Donghu, Wuhan, China | | *A. hantzschii* | |  | |  | | KM514784 | |  | |  |
| 1283-3 | Lake, Donghu, Wuhan, China | | *A. hantzschii* | |  | |  | | KM514781 | |  | |  |
| 1283-4 | Lake, Donghu, Wuhan, China | | *A. hantzschii* | |  | |  | | KM514782 | |  | |  |
| 1283-5 | Pond Guanqiao, Jingzhou, China | |  | |  | |  | | KM514778 | |  | |  |
|  |  | |  | |  | |  | |  | |  | |  |
| **Outgroup** |  | |  | |  | |  | |  | |  | |  |
|  |  | |  | |  | |  | |  | |  | |  |
| ***Chlorotetraedron*** |  | |  | |  | |  | |  | |  | |  |
| SAG 43.81 |  | | *C. incus* | | **KC145512** | |  | |  | |  | |  |
|  |  | |  | |  | |  | |  | |  | |  |
| ***Scenedesmus*** |  | |  | |  | |  | |  | |  | |  |
| 1268-1 | Lake, Donghu, Wuhan, China | | *S. spinosus* | |  | |  | | KM514802 | |  | |  |
| 1268-4 | Lake, Donghu, Wuhan, China | | *S. spinosus* | |  | |  | | KM514794 | |  | |  |
| BCP-SEV3VF4 |  | | *S. rotundus* | | **HQ246351** | |  | |  | |  | |  |
|  |  | |  | |  | |  | |  | |  | |  |
| ***Pleurastrum*** |  | |  | |  | |  | |  | |  | |  |
| DB-1 | Lake Xuanwuhu, Nanjing, China | | *P. insigne* | | KM514861 | |  | |  | |  | |  |
| DB-2 | Lake Taihu, Jiangsu, China | | *P. insigne* | |  | |  | | KM514747 | |  | |  |
| SM2-4 | Soil from Shihezi, Xinjiang, China | | *P. insigne* | | KM514862 | | KM514812 | |  | |  | |  |
| SAG 30.93 |  | | *P. insigne* | | **EF113464** | |  | |  | |  | |  |
