# Supplementary material for: Combining and Comparing Coalescent, Distance and Character-Based Approaches for Barcoding Microalgaes: A Test with Chlorella-Like Species (Chlorophyta)
Source: PLoS One. 2016 Apr 19;11(4):e0153833. doi: 10.1371/journal.pone.0153833 (PMC4841637; doi:10.1371/journal.pone.0153833)
Supplement: S2 Table — (DOC) [file pone.0153833.s013.doc]

**S2 Table.** Primer sequences and annealing temperatures used to amplify the different regions. Names of the forward (-F) and reverse (-R) primers pairs are indicated and also their references.

|  | | | | | | | | |
| --- | --- | --- | --- | --- | --- | --- | --- | --- |
| **Name** | **Sequence 5'–3'** | | | | **Annealing**  **temperature (°C)** | | **Source** | |
| ***rbcL*** |  | | | |  | |  | |
| *rbcL*-F | ATG KCT CCA CAA ACT GAA ACT A | | | | 49-50 | | Sun et al. 2009 | |
| *rbcL*-R | TTA AAG WGT ATC GAT WGT TTC GA | | | | 49-50 | | Sun et al. 2009 | |
| *RcbL*Z-F | CAA CCA GGT GTT CCA SCT GAA G | | | | 49-50 | | This study | |
| *RcbL*Z-R | CTA AAG CTG GCA TGT GCC ATA C | | | | 49-50 | | This study | |
|  |  |  |  |  |  |  |  |  |
| **16S** |  |  | | |  | |  | |
| 359F-T | GGG GAA TTT TCC GCA ATG GG | | | | 47-50 | | Burja et al. 2001 | |
| 781R(b) | GAC TAC AGG GGT ATC TAA TCC CTT T | | | | 47-50 | | Burja et al. 2001 | |
| 16SZ-R | GGT ATC TWA TCC CTT TYG CT | | | | 47-50 | | This study | |
| 16SZ-F | CGC AAT GGG CGA AAG CCT G | | | | 47-50 | | This study | |
|  |  |  |  |  |  |  |  |  |
| **ITS** |  | | | |  | |  | |
| NS7m-F | GGC AAT AAC AGG TCT GT | | | | 56 | | Bock et al. 2011 | |
| LR1850-R | CCT CAC GGT ACT TGT TC | | | | 56 | | Bock et al. 2011 | |
|  |  |  |  |  |  |  |  |  |
| ***tufA*** |  | | | |  | |  | |
| *tufA*F | TGA AAC AGA AMA WCG TCA TTA TGC | | | | 47 | | Famà et al. 2002 | |
| *tufG*F4 | GGN GCN GCN CAA ATG GAY GG | | | | 53 | | Famà et al. 2002 | |
| *tufA*R | CCT TCN CGA ATM GCR AAW CGC | | | | 53 | | Famà et al. 2002 | |
