# Supplementary material for: Combining and Comparing Coalescent, Distance and Character-Based Approaches for Barcoding Microalgaes: A Test with Chlorella-Like Species (Chlorophyta)
Source: PLoS One. 2016 Apr 19;11(4):e0153833. doi: 10.1371/journal.pone.0153833 (PMC4841637; doi:10.1371/journal.pone.0153833)
Supplement: S4 Table — (DOC) [file pone.0153833.s015.doc]

**S4 Table.** Themean interspecific divergences of ITS sequences for *Chlorella-*like taxa (lower left: nucleotide divergences, upper right: standard error), in comparison with the mean intraspecific distance (0.016). The taxa name corresponded to the assignments in Fig 2 and Table 2b.
