# Supplementary material for: Combining and Comparing Coalescent, Distance and Character-Based Approaches for Barcoding Microalgaes: A Test with Chlorella-Like Species (Chlorophyta)
Source: PLoS One. 2016 Apr 19;11(4):e0153833. doi: 10.1371/journal.pone.0153833 (PMC4841637; doi:10.1371/journal.pone.0153833)
Supplement: S5 Table — (DOC) [file pone.0153833.s016.doc]

**S5 Table.** The mean interspecific divergencesof *tufA* sequences for *Chlorella-*like taxa (lower left: nucleotide divergences, upper right: standard error), in comparison with the mean intraspecific distance (0.001). The taxa name according to the assignments in Fig 3 and Table 2c.
